# Human Endogenous Retrovirus (HERV) Transcriptome Is Dynamically Modulated during SARS-CoV-2 Infection and Allows Discrimination of COVID-19 Clinical Stages

**DOI:** 10.1128/spectrum.02516-22

**Published:** 2023-01-05

**Authors:** Nicole Grandi, Maria Chiara Erbì, Sante Scognamiglio, Enzo Tramontano

**Affiliations:** a Laboratory of Molecular Virology, Department of Life and Environmental Sciences, University of Cagliari, Cagliari, Italy; b Istituto di Ricerca Genetica e Biomedica, Consiglio Nazionale delle Ricerche, Cagliari, Italy; Kumamoto University

**Keywords:** SARS-CoV-2, COVID-19, HERV, human endogenous retroviruses, RNA-seq, transcriptome

## Abstract

SARS-CoV-2 infection is known to trigger an important inflammatory response, which has a major role in COVID-19 pathogenesis. In infectious and inflammatory contexts, the modulation of human endogenous retroviruses (HERV) has been broadly reported, being able to further sustain innate immune responses due to the expression of immunogenic viral transcripts, including double-stranded DNA (dsRNA), and eventually, immunogenic proteins. To gain insights on this poorly characterized interplay, we performed a high-throughput expression analysis of ~3,300 specific HERV loci in the peripheral blood mononuclear cells (PBMCs) of 10 healthy controls and 16 individuals being either convalescent after the infection (6) or retesting positive after convalescence (10) (Gene Expression Onmibus [GEO] data set GSE166253). Results showed that the exposure to SARS-CoV-2 infection modulates HERV expression according to the disease stage and reflecting COVID-19 immune signatures. The differential expression analysis between healthy control (HC) and COVID-19 patients allowed us to identify a total of 282 differentially expressed HERV loci (deHERV) in the individuals exposed to SARS-CoV-2 infection, independently from the clinical form. In addition, 278 and 60 deHERV loci that were specifically modulated in individuals convalescent after COVID19 infection (C) and patients that retested positive to SARS-CoV-2 after convalescence (RTP) as individually compared to HC, respectively, as well as 164 deHERV loci between C and RTP patients were identified. The identified HERV loci belonged to 36 different HERV groups, including members of all three classes. The present study provides an exhaustive picture of the HERV transcriptome in PBMCs and its dynamic variation in the presence of COVID-19, revealing specific modulation patterns according to the infection stage that can be relevant to the disease clinical manifestation and outcome.

**IMPORTANCE** We report here the first high-throughput analysis of HERV loci expression along SARS-CoV-2 infection, as performed with peripheral blood mononuclear cells (PBMCs). Such cells are not directly infected by the virus but have a crucial role in the plethora of inflammatory and immune events that constitute a major hallmark of COVID-19 pathogenesis. Results provide a novel and exhaustive picture of HERV expression in PBMCs, revealing specific modulation patterns according to the disease condition and the concomitant immune activation. To our knowledge, this is the first set of deHERVs whose expression is dynamically modulated across COVID-19 stages, confirming a tight interplay between HERV and cellular immunity and revealing specific transcriptional signatures that can have an impact on the disease clinical manifestation and outcome.

## INTRODUCTION

The COVID-19 pandemic has been affecting the global population since December 2019, when the second identified human severe acute respiratory syndrome coronavirus (SARS-CoV-2) gave rise to an epidemic in Wuhan city (Hubei province, China). Since then, the virus has rapidly spread to the rest of the World, leading to around 540 million confirmed cases of COVID-19 and 6.3 million deaths according to the World Health Organization’s dashboard (https://covid19.who.int, accessed on 24 June 2022).

SARS-CoV-2 is a betacoronavirus, belonging to the *Coronavirinae* subfamily of the *Coronaviridae* (order *Nidovirales*). It has a positive-sensed single-stranded RNA (ssRNA+) genome with a 5′ cap and 3′-untranscribed region (UTR) poly(A) tail, which can be directly recognized as an mRNA by the cellular translational apparatus. The SARS-CoV-2 genome is ~30 kb long and includes, in total, 14 open reading frames (ORFs) producing 24 nonstructural proteins (nsp) involved in various steps of viral replication and assembly and 4 structural proteins that constitute the viral components of spikes (S), envelope (E), matrix (M), and nucleocapsid (N) (1). In particular, the first ORF of the genome, namely, ORF1ab, makes up approximately 65% of the viral RNA and is translated into two polyproteins: pp1a (including nsp1 to –11) and pp1ab (including nsp1 to 16 thanks to a readthrough mechanism) (1). These polyproteins include, among the other nsp, SARS-CoV-2 replicative enzymes such as the viral papain-like and the main 3C-like proteases (nsp3-PL^pro^ and nsp5-3CL^pro^, respectively) as well as the RNA-dependent RNA polymerase (nsp12-RdRp).

The infection of SARS-CoV-2 starts with the binding of S protein to the angiotensin-converting enzyme 2 (ACE2) receptor, found on epithelial cells in many mammalian tissues (lower respiratory tract and lungs, but also kidneys, gastrointestinal tract, heart, liver, and blood vessels) (2). Such recognition promotes viral uptake and fusion at the cellular or endosomal membrane, followed by the release and uncoating of SARS-CoV-2 genomic ssRNA+ that is immediately translated at ORF1ab to produce pp1a and pp1ab. The latter are cotranslationally and posttranslationally processed into the single nsp to constitute the viral replication and transcription complex (1). In parallel, the formation of characteristic perinuclear double-membrane vesicles and spherules create a dedicated microenvironment for SARS-CoV-2 RNA replication and expression of subgenomic mRNAs, including the remaining accessory proteins and the structural components of viral particles. The latter are translocated and assembled at the endoplasmic reticulum and Golgi compartment with the new viral genomes, which are already associated with N proteins, and the thus-produced virions finally leave the infected cell by exocytosis (1).

The clinical syndrome associated to SARS-CoV-2 infection, namely, COVID-19, has been intensively studied, revealing a wide range of manifestations that affect not only the primary site of infection, but also show important systemic impairments. Concerning the lungs, clinical signs can be divided into early-phase pathological features (including pulmonary edema, vascular congestion, and inflammatory infiltration with fibrinoid material and multinucleated giant cells) and late-phase manifestations (such as diffuse alveolar injury, acute respiratory distress syndrome, and bilateral inflammatory mononuclear infiltrates) (3). However, the major characteristic of SARS-CoV-2 infection is the outbreak of a potent hyperinflammatory reaction, which can be responsible for multiple organ dysfunction, leading to systemic deterioration and even death or causing permanent and often severe sequelae (3).

Human endogenous retroviruses (HERVs) are genomic relics of ancestral viral infections that threatened primates along their evolution, i.e., in the last 60 million of years (4). All these ancient infectious agents, which included members or all the three classes of retroviruses, have gone extinct, in most cases even before the appearance of the first humans. However, given that their infection and integration targeted the germ line cells, these proviral sequences have been fixed and vertically inherited throughout primates’ evolution, now constituting around the 8% of the human genome as long terminal repeat (LTR)-retrotransposons. Of course, the prolonged persistence in the primate genome exposed HERV sequences to silencing mechanisms and random mutations, leading to the loss of replication capacity. However, a minority of HERVs retain some residual protein-coding potential, and in some instances their products have been domesticated for pivotal physiological functions in the host (4, 5). The latter case includes the cooption of HERV Env fusogenic activity for placenta formation (4), the contribution of ancient retroviral Gag proteins to human brain development and architecture (6), and intriguingly, a pivotal role of the dispersion of endogenous retroviruses (ERV) integrations in the evolution and shaping of innate antiviral immunity networks in mammals (7).

In addition to residual protein production, growing evidence shows that many HERV sequences (even if highly defective) can still have an impact on the host through their regulatory signals, which may influence the expression of nearby cellular genes, and especially, their abundant production of RNA transcripts. The latter, beside the frequent noncoding nature, can impact cellular systems because they include viral signatures that may still be recognized as pathogen-associated molecular patterns (PAMPs) by innate immune pathways (5). For example, it has been shown that transcripts expressed by these multicopy elements can form double-stranded RNA (dsRNA) by complementary pairing, a known PAMP promptly recognized by cellular and endosomal pattern recognition receptors (PRRs), being the first-line sensors of antiviral responses (5). Such signaling triggers a cascade that leads to the nuclear induction of the interferon-I (IFN-I) pathway, with the production of inflammatory cytokines thought to be involved in the manifestation of various noninfectious disorders, including neuroinflammatory diseases and several cancers (4, 5, 8). Intriguingly, the same immunogenic action, detrimental in pathological conditions, is currently being exploited for innovative immunotherapies (9).

Remarkably, if on the one side HERV expression can trigger the host innate immunity, the latter is itself able to modulate HERV expression, establishing a sort of vicious circle. For example, the mimicking of a microbial infection through *in vivo* administration of bacterial lipopolysaccharides (LPS) was shown to differentially modulate the peripheral blood mononuclear cell (PBMCs) expression of 4,607 HERV and mammalian apparent LTR retrotransposon (MaLR) sequences, which showed a general colocalization with cellular genes involved and/or modulated in the innate immune response (10). Similarly, adaptive immune responses evoked by antiviral vaccination have also been shown to influence HERV expression, showing a dynamic modulation of HERV transcripts according to the different stages of antibody production (11). In addition, even with lower genetic resolution, several other studies reported the general modulation of individual HERV group expression in the presence of several viral infections, including among others, HIV (12–15), hepatitis C virus (HCV) (16), influenza (17), and herpesviruses (18–21).

Indeed, to date, very few reports have assessed the transcriptional modulation of HERV loci by SARS-CoV-2 infection or, in light of the predominant inflammatory environment, their specific pattern of expression according to COVID-19 clinical stages and disease severity (22–24). Such interplay is highly relevant, especially considering that HERV transcriptional activation may sustain and amplify the inflammatory activation exerted by COVID19, which is a major cause of severe symptoms and death, as well as of permanent sequelae in convalescent patients.

In the present study, we performed a high-throughput analysis of the expression of about 3,300 HERV loci in the peripheral blood mononuclear cells (PBMCs) from individuals convalescent after COVID-19 infection (C, *n* = 6) and patients that retested positive to SARS-CoV-2 after convalescence (RTP, *n* = 10) compared to healthy controls (HC, *n* = 10) from the transcriptome sequencing (RNA-seq) data set recently published by Wang and coworkers (Gene Expression Onmibus [GEO] accession no. GSE166253) (25). Briefly, C patients were defined as patients that have been discharged from the hospital based on (i) normal body temperature for >3 days, (ii) at least two consecutive negative SARS-CoV-2 reverse transcriptase quantitative PCR (RT-qPCR) assays, (iii) improved respiratory symptoms, and (iv) significant absorption of pulmonary lesions. RTP were C individuals that turned positive at follow-up after discharge from the hospital (≥2 consecutive RT-qPCR assays) (25). In the original study, blood sampling for PBMC isolation and RNA-seq were performed 2 to 4 weeks after discharge (C) or immediately after the novel positivity confirmation (RTP). Neither C nor RTP patients showed severe COVID-19 manifestations (25). The analysis of their transcriptomes in the present study allowed us to identify a total of 282 differentially expressed HERV loci (deHERV) in individuals exposed to SARS-CoV-2 infection (RTP and C, considered together) compared to HC. Of these deHERVs, 202 were also found to be significantly modulated in the subcomparisons specific to each clinical stage (RTP versus HC and C versus HC). In addition, these subcomparisons allowed us to identify 278 and 60 deHERV loci that were specific to the stage of C and RTP patients individually compared to HC, respectively, while 22 other deHERV sequences were modulated only when the two clinical stages were analyzed excluding HC (RTP versus C). Finally, 31 HERV loci were significantly modulated in all analyses, showing a dynamic transcriptional shift across COVID-19 clinical stages and were characterized in greater detail.

## RESULTS

### HERV transcriptome is globally influenced by SARS-CoV-2 exposure.

**FIG 1:**
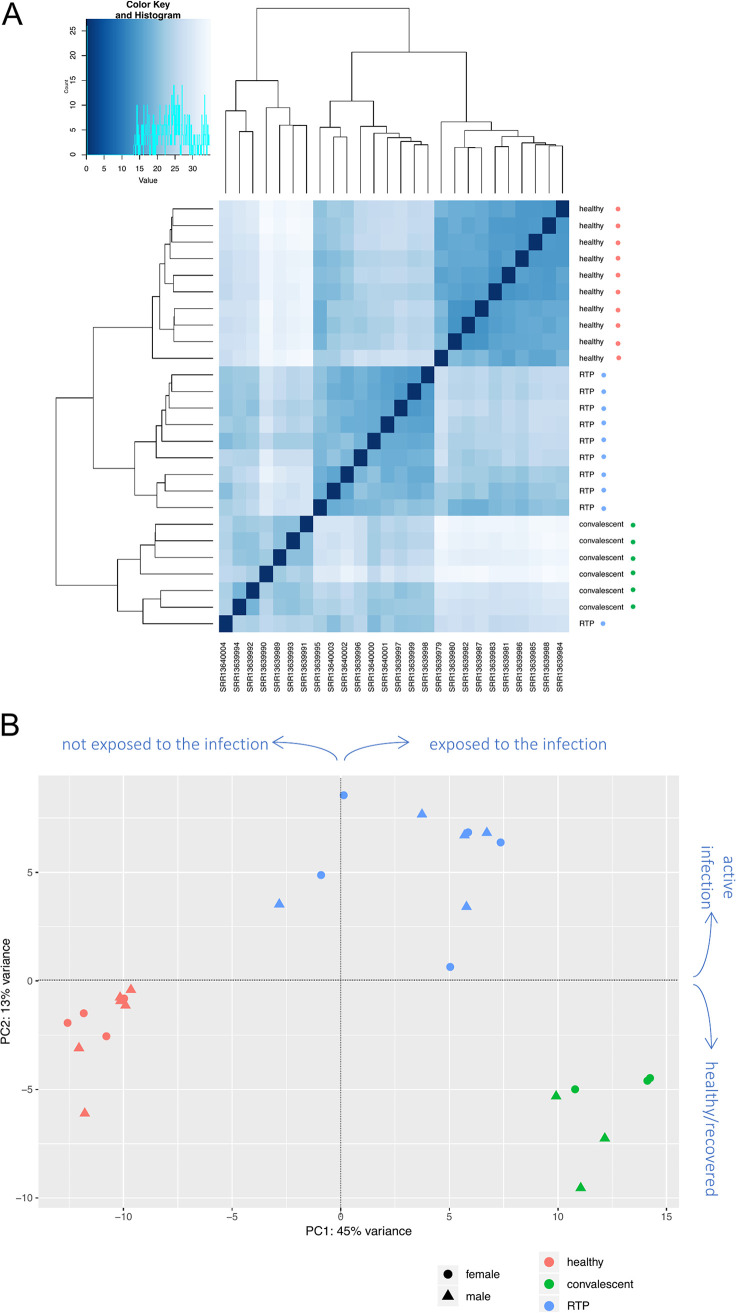
HERV sample-to-sample distance (A) and PCA plot (B). (A) Heatmaps of the overall similarity between samples: the correlation distance measure was used in clustering columns based on the rlog-normalized HERV expression data. Samples are annotated by condition: red, healthy controls; green, convalescent after recover from SARS-CoV-2 infection; blue, retesting positive after convalescence. The three clusters highlight specific HERV transcriptional signatures induced by SARS-CoV-2 exposure and infection. Distance values are shades of blue, as represented in the color key and histogram legends. (B) Principal-component analysis as performed on rlog-normalized HERV loci expression data. It is possible to see the division between nonexposed controls and individuals exposed to SARS-CoV-2 infection according to the PC1 (45% of variance) as well as the division according to the presence of SARS-CoV-2 active infection by the PC2 (13% of variance).

At first, to assess whether the presence of SARS-CoV-2 infection could have an impact on the overall HERV expression, we performed clustering analyses. Results shown in the sample-to-sample distance heatmap (Fig. 1A) demonstrated that the general expression of HERV sequences is strongly influenced by the presence of SARS-CoV-2, allowing us not only to distinguish healthy controls (HC) from SARS-CoV-2-exposed individuals, but even to discriminate between past exposure (C) and active reinfection (RTP).

To gain further insights about the impact of SARS-CoV-2 infection on HERV transcriptional variance among the three conditions, we performed an unsupervised principal-component analysis (PCA) (Fig. 1B). Such analysis evaluates HERV expression among samples and distributes the latter in a bi-dimensional matrix to identify the first and second principal components (PC1 and PC2, respectively) accounting for its variation. The analysis confirmed a major impact of SARS-CoV-2 infection on HERV transcriptional variation (Fig. 1B). In fact, samples were divided in three clusters based on HERV transcriptional variation, corresponding to the three conditions (HC, C, and RTP), and both the PC1 and the PC2 appeared to be linked to SARS-CoV-2 infection stage. In particular, the PC1 accounted for 45% of the total variance in HERV expression and broadly divided HC from C and RTP patients, corresponding to the exposure to SARS-CoV-2 infection (Fig. 1B), while the PC2 was responsible for an additional 13% of HERV transcriptional variance and divided the cluster of RTP patients, i.e., active reinfections, from the SARS-CoV-2-negative individuals, either because they were never infected (HC) or already recovered (C).

The same clustering analyses were performed considering the transcriptional activity of cellular genes (see Fig. S1 in the supplemental material). As expected, SARS-CoV-2 infection has a global modulatory effect on cellular gene expression as well, dividing the three groups of individuals in the sample-to-sample distance plot (Fig. S1A). However, in this case the presence of the infection had a lower impact on the cellular gene transcriptional activity and did not seem to be a principal component for its variance (Fig. S1B). In fact, samples in this case were divided by PC1 (54%) in two dispersed groups, RTP (2/10) and C (6/6) individuals on the one side and RTP (8/10) ad HC (10/10) on the other side, while PC2 (9%) divided samples into two mixed groups, both including individuals of the three conditions (Fig. S1). To understand whether the different transcriptional patterns as observed with cellular genes could be linked to a basal different response to innate immune stimuli, we considered a subset of 44 genes capable of deconvoluting complex responses to immune stimulation, distinguishing in this way low from high responders (26). Sample-to-sample distance analysis performed on these 44 immune genes showed in this case a different distribution, grouping the samples in three clusters, with clusters 2 (6 RTP and 2 C) and 3 (4 C) showing very similar transcriptional signatures compared to cluster 1 (10 healthy and 4 RTP) (Fig. S2A). Such a division also resembled the distribution observed in the PCA built from the whole genic data set, suggesting that—for cellular gene expression—a major variant is represented by the basal, individual immune activation (Fig. S1B). Accordingly, the PCA built with the 44 immune genes perfectly matched the sample-to-sample distance division of the same, suggesting that the PC1 (65%) for cellular gene expression is represented by the different signature of induced cytokine response, dividing low responders (cluster 1, in which some RTP are comparable to “unstimulated” HC samples) from high responders (clusters 2 and 3) (Fig. S2B).

### COVID-19 stages are associated with specific transcriptional modulation of HERVs.

**FIG 2:**
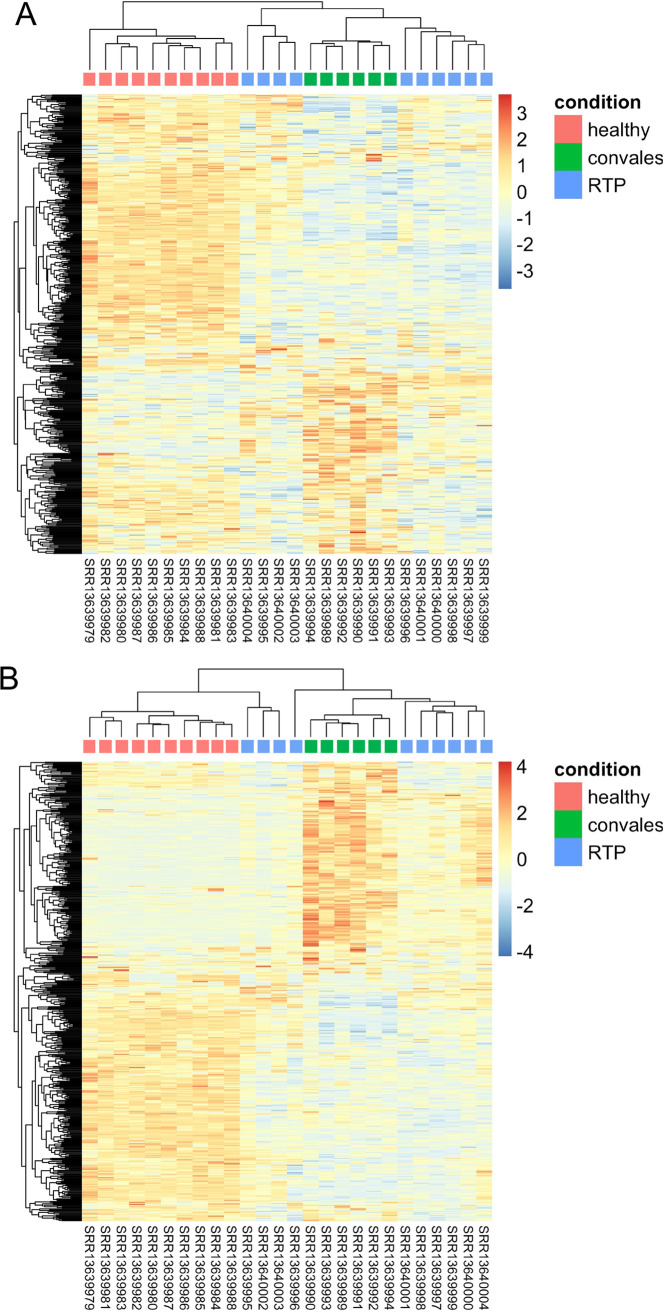
(A and B) Heatmaps based on the top 500 HERVs as sorted by the highest mean (A) or variance (B) of expression. Hierarchical clustering of the top 500 HERV insertions with the highest average (A) or variance (B) of rlog-normalized counts. The top 500 HERV loci are in rows, and the samples are in columns. rlog-normalized counts are color-scaled from blue (minimum) to red (maximum). The correlation distance measure was used in clustering columns. Samples are annotated by condition: red, healthy controls; green, convalescent after recover from SARS-CoV-2 infection; blue, retesting positive after convalescence.

Given that the HERV transcriptome showed specific patterns of modulation according to both the presence and absence of the infection and the different clinical stages, we investigated in more detail the expression of individual HERV sequences in the three groups through the generation of expression heatmaps. The latter were built with the 500 HERV loci showing either the highest mean or variance of expression among samples (Fig. 2A and B, respectively). In both heatmaps, HERV expression was confirmed to show major variations according to the exposure to SARS-CoV-2 infection and COVID-19 clinical stages, clearly clustering the samples according to their condition of HC and recovered (C) or actively infected patients (RTP). Accordingly, these three groups showed different modulation patterns for the same subset of HERVs (Fig. 2A and B, respectively). In addition, RTP patients were divided in two clusters that showed a different localization based on the type of HERV selection (i.e., by either highest mean or variance of expression). While the top 500 HERVs sorted by mean of expression showed a transcriptional behavior comparable to the one of C patients for both RTP clusters (Fig. 1A), the selection of the top 500 HERVs based on the higher variance led 1 of the 2 RTP clusters (*n* = 3) to group with HC (Fig. 2B). A similar clustering was observed in the corresponding heatmaps as generated with the top 500 cellular genes, similarly sorted by the highest mean or variance of expression (Fig. S3A B, respectively). Overall, this analysis confirmed that SARS-CoV-2 exposure has a major impact on HERV expression, leading to specific transcriptional patterns associated with HC, C, and RTP conditions. The fact that a similar behavior was shown with the same selection on cellular genes suggests that the indirect impact of SARS-CoV-2 infection on the PBMC transcriptome is a major determinant of transcriptional modulation, influencing the expression of both canonical genes and endo-retroviral loci.

### Exhaustive analysis of HERV modulation by SARS-CoV-2 exposure.

In light of the above-described results, we wanted to identify the individual HERV loci whose expression is significantly modulated in the presence of SARS-CoV-2 infection, and hence, we performed a differential expression analysis on the whole data set, setting a double statistical threshold (adjusted *P* value [*P*-adj]of ≤ 0.01 and absolute log_2_ fold change of ≥1).

**FIG 3:**
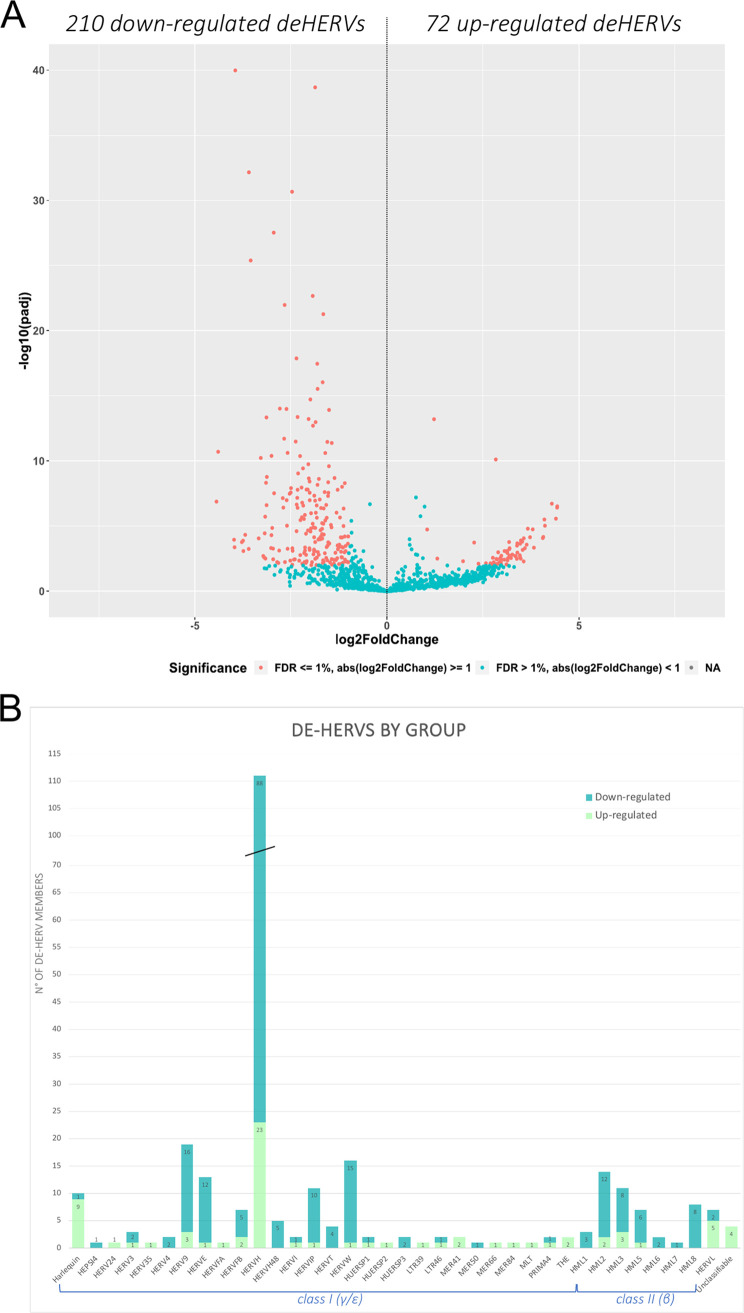
Results of HERV differential expression analysis on the total data set. (A) Volcano plot in which each point represents an individual HERV locus, which spread according to the magnitude (log_2_ fold change, *x* axis) and statistical significance (log_10_ -adjusted *P* values, *y* axis) of its modulation in SARS-CoV-2-exposed individuals (RTP and C) compared to healthy controls (HC). Red points indicate significantly modulated HERVs. (B) Summary of the number of deHERVs as divided by group: both upregulated and downregulated members are reported.

This allowed us to identify a total of 282 deHERVs compared to HC, with *P*-adj values of 0.01 to 1.05^−40^ (Table S2). These deHERVs were further divided according to their magnitude of change in 72 upregulated HERVs (*P*-adj values of 0.01 to 6.22^−14^, log_2_ fold change from of 1.05 to 4.43) and 210 downregulated HERVs (*p*-adj values of 0.01 to 1.05^−40^, log_2_ fold change of –1.01 to –4.44) (Fig. 3A, and Table S2). Among the deHERVs, the majority belong to class I HERV groups (225/282, 80%), half of which belong to the HERV-H group (111/282, 39%) (Fig. 3B). This result is in line with the fact that class I HERVs are more abundant among the 3 classes, with HERV-H being the most numerous. Among the other 20% of deHERVs, 16% belong to the class II HERV-K supergroup (46/282), including members from HML1 to 3 and from HML5 to 8, while the remaining 4% are divided between the class III HERV-L group (7/282) and unclassified elements (4/282), based on the previous classification using ReTe software (27).

For completeness, we performed the same differential expression analysis for the Gencode set of cellular genes, also considering that their modulation could influence the expression of colocalized HERV elements. Among the ~60,700 cellular genes included in the study, 1,373 (2%) were upregulated and 2,643 (4%) were downregulated in individuals exposed to COVID-19 infection compared to HC (data not shown). Their colocalization with deHERVs and the possible impact on their transcriptional modulation analyzed in the following section.

### Characterization of the integration sites of deHERVs.

**FIG 4:**
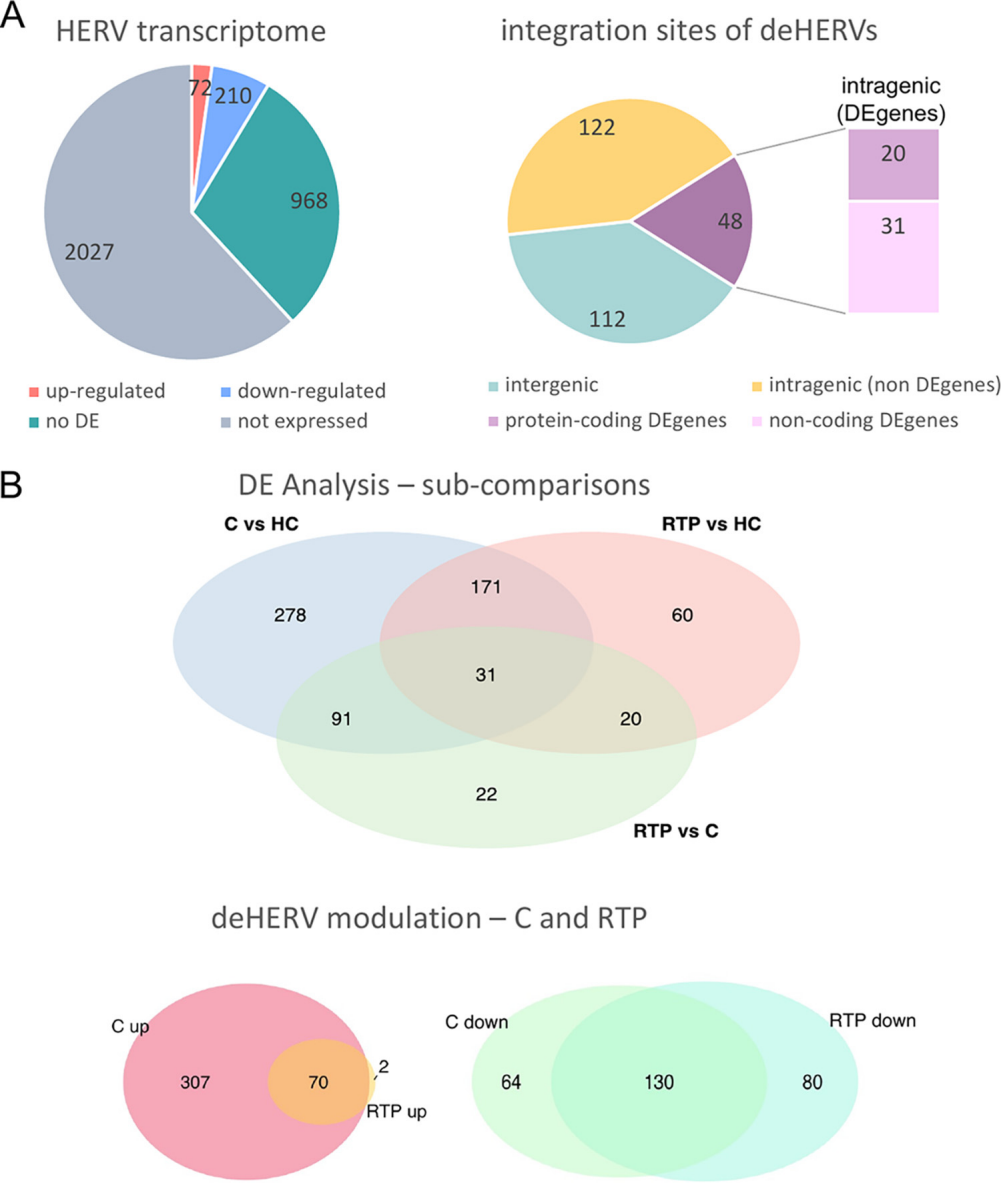
Details of HERV modulation in the overall analysis and in the different subcomparisons. (A) Left: diagram representing the results of the overall differential expression analysis (RTP and C versus HC) in terms of number of HERVs found to be differentially expressed (being either up- or downregulated), expressed but not differentially expressed (no-DE), and not expressed at all. Right: genomic context of integration of the deHERV sequences, which were found either in the intergenic position or within a gene; the latter were further distinguished since the colocalized gene was itself DE and, in this case, if it was coding a protein or not. (B) Venn diagrams of the different differential expression subcomparisons. Top: results and overlaps of the DE analysis as performed in C versus HC, RTP versus HC, and RTP versus C; bottom: detail of the number of deHERVs showing a concordant modulation in C and RTP individually compared to HC.

To further investigate HERV modulation after SARS-CoV-2 exposure, we analyzed the genomic context of insertion of each deHERV to assess their colocalization with cellular genes. Among the 282 deHERVs, 170 (~60%) were colocalized with a total of 205 cellular genes that were either protein-coding (110) or not (95) (Table S3, Fig. 4A). Among the 205 genes that held deHERV integrations, only a minority (50 genes, 24%) was itself modulated by the infection, including 19 protein-coding genes and 31 non-protein-coding genes that were colocalized with 48 deHERVs (Table S3, Fig. 4A). We then compared the modulation of these 50 de-genes with that of the respective colocalized deHERV, observing a concordant modulation in all cases except for the deHERV locus 2328 (HERV-H) (Table S4). The latter was downregulated in RTP and C individuals, while the surrounding gene (RNF217 antisense RNA 1, RNF217-AS1) was upregulated under the same conditions. The rest of the de-genes were upregulated (8) or downregulated (41) concomitantly with the corresponding deHERVs (Table S4, Fig. 4A). We assessed the function of the 8 upregulated de-genes to check if they are involved in innate immune pathways, which would explain their activation in RTP and C patients. While 2 of them were non-protein-coding genes with unknown roles, the other 6 upregulated de-genes included 4 genes involved in immune responses: COLEC12 (collectin subfamily member 12, colocalized with a MER41 element) is a scavenger receptor that binds carbohydrate antigens on microorganisms, facilitating their recognition and removal; TNFRSF21 (tumor necrosis factor [TNF] receptor superfamily member 21, colocalized with an HML3 element) activates nuclear factor kappa-B and mitogen-activated protein kinase 8 (NF-κB and MAPK8, respectively) and plays a role in T helper cell activation, inflammation, and immune regulation; PKHD1 (ciliary IPT domain-containing fibrocystin/polyductin, colocalized with a HERV-H element) can control the signal transducer and activator of transcription 3 (STAT3) signaling, which in turn, plays a role in regulating the host response to viral and bacterial infections; and PRKAR2B (protein kinase cAMP-dependent type II regulatory subunit beta, colocalized with a MER66 element) is one of the 4 regulatory subunits of the cAMP-dependent protein kinase, which can be phosphorylated by the activated catalytic subunit and suppress the transcriptional activity of the cAMP responsive element binding protein 1 (CREB1) in activated T cells. The remaining 2 upregulated de-genes had different activities: FLVCR2 (feline leukemia virus subgroup C receptor 2, colocalized with a HERV-H element) is a retroviral-like transmembrane Ca transporter that may play a role in the development of brain vascular endothelial cells, while PRRG1 (proline rich and Gla domain 1, colocalized with another HERV-H element) encodes a vitamin K-dependent transmembrane protein (Table S4). Analysis of the 41 downregulated de-genes showed that most of them (28) were non-protein coding (Table S4), including also a retroviral gene from a HERV-48 locus in chromosome 21 (ERVH48-1). The remaining 13 downregulated coding genes encompassed, among others, a HERV gene in chromosome 7 (ERV3-1, obviously colocalized with the corresponding HERV-3 sequence 2521) that encodes an Env protein. This HERV is colocalized also with ZNF117, a cellular gene containing multiple zinc finger motifs, which is also downregulated (Table S4).

Overall, even if the majority of deHERV were colocalized with cellular genes, the latter were usually not differentially expressed or showed no apparent transcriptional impact on HERV expression, further suggesting a direct effect of SARS-CoV-2 exposure on their modulation.

### Identification of deHERV signatures specific to COVID-19 clinical stages.

We then asked whether some specific HERV transcriptional signature could be found in RTP and C patients, beside the already reported differences compared together to healthy, unexposed controls. To this purpose, we conducted the differential expression analysis for all the relevant subcomparisons: C versus HC, RTP versus HC, and RTP versus C (Table S5, Fig. 4B). The 282 deHERVs, as identified in the global analysis (of which 72 were upregulated and 210 were downregulated in exposed individuals; Table S2), were the same as those that were differentially expressed in the RTP versus HC subcomparison, showing a concordant modulation with the former results in all cases (Table S5).

Indeed, the C versus HC subcomparison showed a total of 571 deHERVs, of which 377 were upregulated and 194 downregulated in C individuals (Table S5). Of these, 202 (35%) were in common with RTP versus HC subcomparison, showing a concordant modulation in all cases except for 2 HERV loci that were upregulated in C individuals and downregulated in RTP subjects (Table S5, Fig. 4A and B). Of note, the 202 C-RTP-shared deHERV loci included, overall, the 97% and 62% of the upregulated and downregulated deHERV loci found in RTP individuals, respectively.

Lastly, the RTP versus C subcomparison was conducted excluding the HC condition to assess significant differences in the HERV transcriptional pattern according to the sole clinical stage (Table S5, Fig. 4B). The differential expression analysis between RTP and C conditions identified 164 deHERVs, of which 51 (31%) were also found to be modulated in the overall comparison (C and RTP versus HC). Among these, 31 were also shared by both RTP and C subsets compared individually to HC, while the other 20 were specifically modulated under the RTP condition (Fig. 4B). The remaining 119 deHERVs (69%) were either specifically modulated under the C condition (91) or exclusively found as modulated when comparing RTP to C conditions, but not with respect to HC (Fig. 4B). The analysis of the effects of the modulation showed that 45 deHERVs were upregulated and 119 were downregulated: this result refers to RTP compared to C, meaning that the 45 upregulated HERVs show a significantly increased expression in RTP state, while the 119 downregulated HERVs show a significantly increased expression in C state (Table S5, Fig. 4B).

To our knowledge, this is the first set of deHERVs whose expression is dynamically modulated across COVID-19 clinical stages, revealing that past exposure to COVID19 triggers a delayed and wider transcriptional modulation of HERV elements in C individuals compared to actively infected ones, with 369 additional HERV loci that become modulated (being upregulated in most cases) and only a minority that return instead to the original, “basal” condition.

Finally, given that a proportion of the deHERVs as identified in the three above-described subcomparisons were colocalized with de-genes, we investigated the related biological processes using gene ontology (GO) to further characterize those de-genes that were upregulated and colocalized with upregulated deHERVs, i.e., showing a del+/del+ pattern (Fig. S4, Table S6). The latter included 8, 48, and 23 de-genes in RTP versus HC, C versus HC, and RTP versus C subcomparisons, respectively. GO analysis showed that del+/del+ genes were associated with the impairment of cellular immune effectors in the PBMCs of RTP individuals, with the two most significant GO processes represented by the negative regulation of interleukin-13 (GO:0032696) and interleukin-5 (GO:0032714) production in the case of RTP versus HC (*P* = 0.0020, TNFRSF21 gene) and the negative regulation of immunoglobulin production (GO:0002638) and B cell differentiation (GO:0045578) in the case of RTP versus C (*P* = 0.0057, CR1 gene) (Fig. S4, Table S6). Besides these processes, additional immune pathways were affected with lower significance in RTP cells by the same genes. In RTP PBMCs compared to HC, B cell proliferation (GO:0030889) was also negatively regulated, along with an increase of the lymphocyte apoptotic process (GO:0070227) (*P* = 0.0048 and *P* = 0.0024, TNFRSF21 gene) (Table S6). The RTP versus C comparison identified even more affected pathways, highlighting the negative regulation exerted by CR1 on complement activation (GO:0045959 and GO:0045916, with *P* = 0.0091 and 0.0137, respectively) on the production of immune response mediators (GO:0002701, *P* = 0.0103), and in the humoral response mediated by circulating immunoglobulins (GO:0002924, *P* = 0.0114) (Table S6).

### Characterization of 31 key deHERVs showing dynamic modulation across COVID-19.

**FIG 5:**
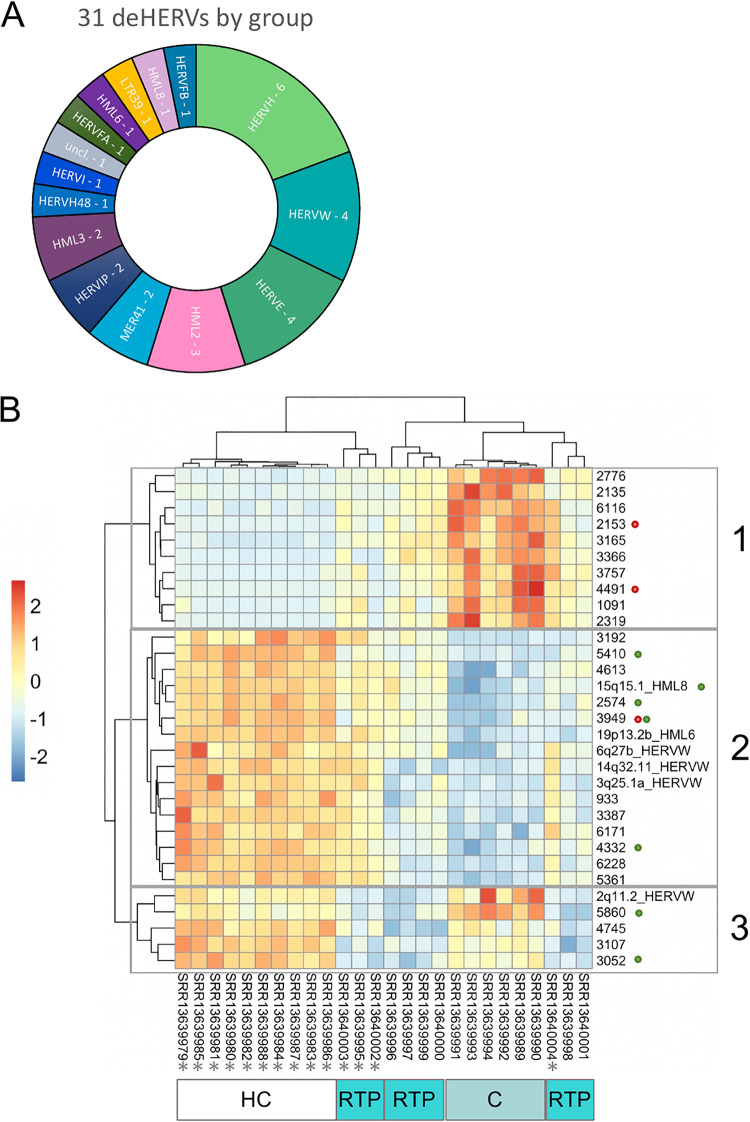
The 31 key deHERVs showing a significant modulation in all DE analyses. (A) Distribution among the different HERV groups. (B) Heatmap of expression based on the calculation of transcripts per million kilobases values (TPM); the concomitant modulation of eventual colocalized de-genes, when present, is indicated with red and green dots, meaning colocalization with an upregulated or downregulated gene(s), respectively. Samples predicted to be low responders based on the analysis of the 44 key innate immune genes are marked with an asterisk (*).

We then focused our attention on the 31 key deHERVs that showed significant modulation in all the comparisons (C versus HC, RTP versus HC, RTP versus C) to assess their behavior under the different conditions (Table 1, Fig. 5). The 31 key deHERVs belonged to 14 different HERV groups, including both class I and class II representatives (11 and 3 groups, respectively), plus an element with uncertain classification (Fig. 5A). Concerning the modulation, most of them were downregulated or upregulated in both C and RTP individuals compared to HC (*n* = 19 and 10, respectively), while 2 showed a discordant modulation between the two categories of SARS-CoV-2-exposed individuals, being upregulated under the C condition only (Table 1). In addition, 9 of these deHERVs were colocalized with genes that were themselves modulated in the overall analysis (C and RTP versus HC), usually showing concordant behavior (Table 1).

**TABLE 1:** Results for the 31 key deHERV loci that showed significant modulation in all analyses^*a*^

31 Common deHERVs		Results from RTP vs C			Results from C vs HC			Results from RTP vs HC			Colocalized de-genes (results from global comparison)				
ID	Group	Log_2_ FC	*P*-adj	del2	Log_2_ FC	*P*-adj	del2	Log_2_ FC	*P*-adj	del2	Gene name	Prot. coding	del2	Log_2_ FC	*P*-adj
2776	HERVE	–2.5	1.15E-22	DEL −	3.5	1.31E-43	DEL +	1	1.83E-05	DEL +					
2135	HML3	–4.6	9.08E-20	DEL −	6.9	2.01E-31	DEL +	2.3	0.0001827	DEL +					
2q11.2	HERVW	−3	2.84E-15	DEL −	1.1	0.0036475	DEL +	−1.9	4.63E-08	DEL −					
5860	HERVFB	−3.3	4.18E-15	DEL −	1.4	2.40E-05	DEL +	−1.9	1.38E-05	DEL −	AL591845.1	no	DEL −	−1.21	0.009788
4613	HERVH	1.9	9.15E-10	DEL +	−3.1	3.99E-25	DEL −	−1.2	9.67E-09	DEL −					
15q15.1	HML8	1.6	3.51E-09	DEL +	–2.7	1.46E-26	DEL −	–1.1	4.54E-07	DEL −	RAD51-AS1	no	DEL −	−1.13	1.42E-07
2574	HERVE	1.9	1.16E-08	DEL +	–3.5	1.02E-28	DEL −	−1.6	2.46E-11	DEL −	AC079781.5	no	DEL −	−1.21	1.92E-06
3052	HERVH	–1.7	3.31E-07	DEL −	−1.3	3.64E-06	DEL −	–2.9	2.96E-28	DEL −	AL355377.4	no	DEL −	−2.79	2.46E-11
6228	HERVIP	2.9	1.98E-06	DEL +	–5.4	2.74E-21	DEL −	−2.5	1.22E-08	DEL −					
3107	HERVH	–1.2	4.54E-06	DEL −	−1.3	1.94E-07	DEL −	−2.5	2.15E-31	DEL −					
6171	HML2	1.5	2.83E-05	DEL +	−2.7	5.19E-16	DEL −	−1.2	7.31E-05	DEL −					
6116	LTR39	−2.2	3.03E-05	DEL −	5.3	2.22E-11	DEL +	3.2	0.0001674	DEL +					
3192	HML2	4.6	4.92E-05	DEL +	−6.3	1.80E-09	DEL −	−1.8	0.0015817	DEL −					
6q27b	HERVW	1.7	0.000101	DEL +	−2.8	6.48E-12	DEL −	−1.1	0.0010291	DEL −					
5361	HERVE	1.5	0.000108	DEL +	−2.7	2.16E-14	DEL −	−1.2	2.19E-06	DEL −					
3387	HERVIP	2.5	0.000139	DEL +	–4.6	6.85E-15	DEL −	−2.2	2.84E-06	DEL −					
1091	MER41	–2.4	0.00015	DEL −	5.2	9.49E-10	DEL +	2.8	0.0027002	DEL +					
19p13.2b	HML6	2	0.000165	DEL +	−3.8	1.87E-15	DEL −	−1.8	2.61E-07	DEL −					
3165	HERVFA	–1.9	0.000226	DEL −	6.2	1.64E-14	DEL +	4.3	1.90E-07	DEL +					
4491	MER41	–2.7	0.000548	DEL −	5.5	1.87E-09	DEL +	2.8	0.0059368	DEL +	COLEC12	Yes	DEL +	1.99	0.006223
3949	HERVE	1	0.000575	DEL +	–2.8	2.70E-26	DEL −	–1.8	2.95E-16	DEL −	AL590064.1	No	DEL −	−1.31	3.24E-08
											FLVCR2	Yes	DEL +	2.37	2.39E-08
2153	HML2	−2.5	0.000627	DEL −	6.2	8.28E-12	DEL +	3.7	7.13E-05	DEL +	TNFRSF21	Yes	DEL +	2.08	6.27E-06
4332	HML3	1.1	0.000803	DEL +	−2.6	1.04E-18	DEL −	−1.5	4.31E-09	DEL −	AC108134.3	No	DEL −	−1.28	7.85E-07
2319	Unclassifiable	−2.3	0.001278	DEL −	5.4	5.66E-10	DEL +	3.1	0.0008136	DEL +					
4745	HERVH	−1.4	0.001808	DEL −	−1	0.0088384	DEL −	−2.4	3.23E-12	DEL −					
3q25.1a	HERVW	3.5	0.002572	DEL +	–5.4	5.59E-07	DEL −	−1.8	0.0026058	DEL −					
3366	HERVH	–1.9	0.002919	DEL −	6	2.58E-12	DEL +	4.1	3.11E-06	DEL +					
3757	HERVI	−2	0.00398	DEL −	5.4	9.41E-10	DEL +	3.4	0.0002105	DEL +					
5410	HERVH48	2.5	0.006516	DEL +	−6.1	1.85E-14	DEL −	−3.6	6.91E-33	DEL −	Z83745.1	No	DEL −	−3.64	8.28E-25
933	HERVH	1.5	0.006929	DEL +	–3.7	3.60E-14	DEL −	−2.2	1.60E-07	DEL −					
14q32.11	HERVW	1.9	0.007315	DEL +	–3.3	2.36E-07	DEL −	−1.4	0.0039035	DEL −					

a The table shows the results of differential expression analysis as performed in pairwise comparisons of the expression between RTP and C SARS-CoV-2-exposed individuals, C and HC, and RTP and HC. The results for the eventual colocalized genes, when significantly modulated, are also reported and refer to the overall comparison (C and RTP individuals versus HC). FC, fold change; del2, the observed modulation: DEL+ indicates upregulation while DEL− indicates downregulation; prot., protein.

To gain more insights on these 31 key deHERVs and the biological relevance of their transcriptional activity, we used the raw read counts to calculate their expression values as transcripts per million kb (TPM) (Table S7). We chose TPM normalization because it allows us to compare the expression levels of sequences of different lengths, considering the sequencing depth of the various samples as well. Results from the TPM calculation for the 31 key deHERVs under each condition are depicted in Fig. 5B and clearly show the transcriptional behavior of the same HERV under the different conditions. According to the TPM values, the 31 deHERVs were divided by the dendrogram in the *y* axis into three main clusters, highlighted with gray boxes (Fig. 5B). The upper cluster (indicated as no. 1 in the figure) included 10 deHERVs with a higher expression in C and a lower expression in HC. The middle cluster (no. 2) showed an opposite trend, including 16 deHERVs with a higher expression in HC and a lower expression in C. In both these clusters, RTP showed an intermediate modulation of the deHERVs. The lower cluster (no. 3) included 5 deHERVs with a higher and comparable expression in C and HC, respectively, and a lower one in RTP. In the same heatmap, the samples were clustered based on the deHERVs’ TPM, as represented by the dendrogram in the *x* axis (Fig. 5B). Of note, while all HC and C samples were clustered according to their condition, forming two homogeneous groups, RTP samples were split into three clusters. While two of these RTP clusters were related to the C cluster, the first was more closely related to HC based on the expression of a subset of 9 deHERVs (Fig. 5B). A closer look revealed that this cluster includes 3 of the 4 individuals that were predicted to be low responders based on the analysis of the 44 key immune genes, in which they were grouped with HC samples as well (Fig. S2).

**FIG 6:**
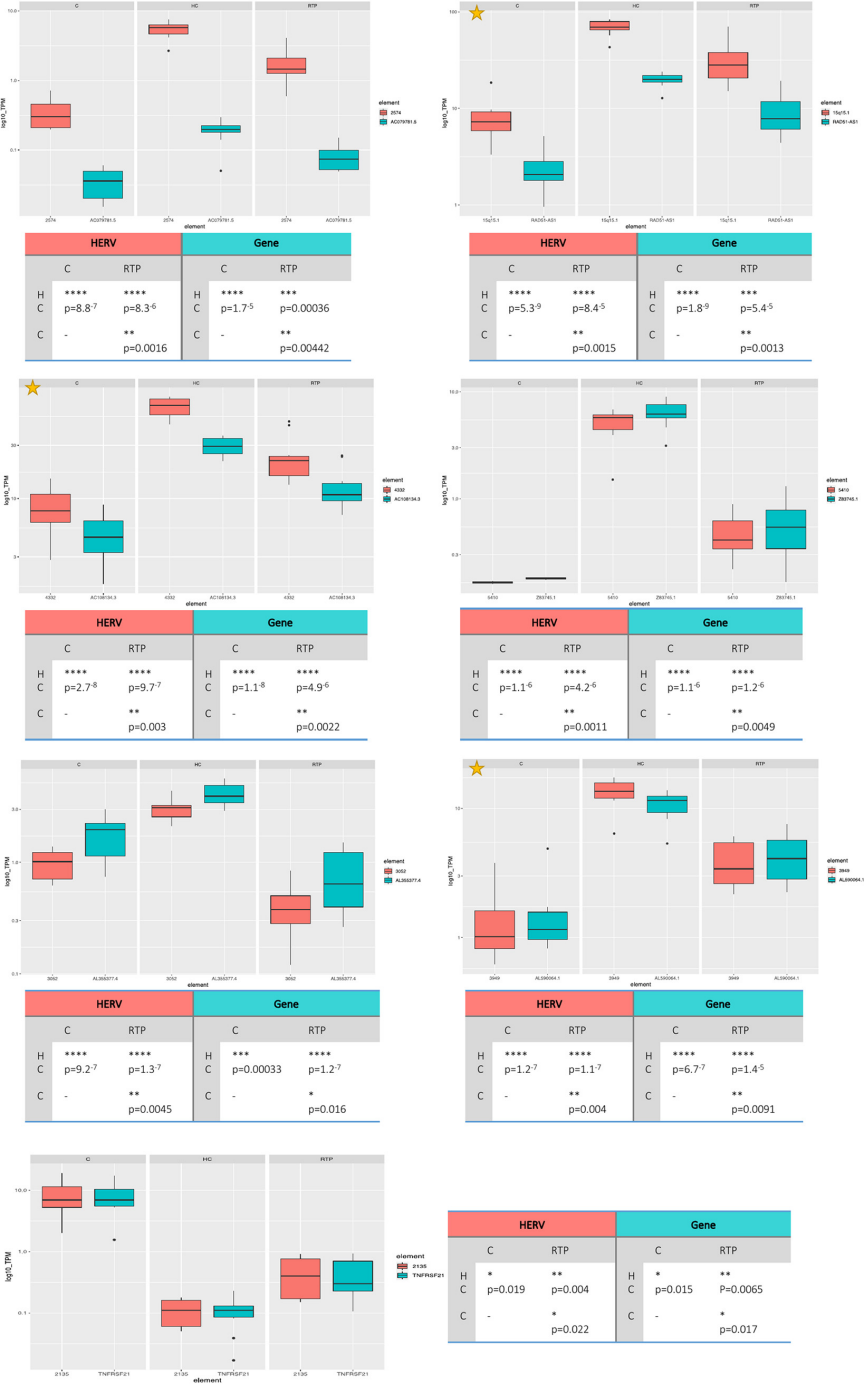
Boxplot of expression levels for the key deHERVs modulated in all conditions that are colocalized with de-genes. The expression levels (as transcripts per million kilobases values [TPM]) of the 7 out of 31 key deHERVs integrated within cellular genes that were themselves modulated are plotted along with those of the colocalized genes under the different conditions, to assess the possible reciprocal influence. Plots marked with an asterisk are the deHERVs with the highest expression (log_10_ TPM, >10 for at least one condition). Statistics are based on *t* test.

Finally, we filtered the 31 deHERV loci based on a TPM threshold, selecting the 20 showing a mean TPM of >2.5 under at least one condition (Table S7). Overall, the highest TPM values were observed in HC (maximum [max], 109.9; mean, 14.6, median, 4.2), followed by RTP (max, 79.8; mean, 5.8; median, 1.3) and C (max, 26.6; mean, 3.1; median, 0.6), based on the fact that most of them were downregulated in the individuals exposed to SARS-CoV-2. TPM values of the 20 selected deHERVs were then plotted and statistically analyzed between conditions: the results are shown in Fig. 6 for the 6 deHERV loci colocalized with cellular genes that were themselves modulated in the overall analysis (C and RTP versus HC) and in Fig. S5 for the remaining 14 deHERVs. The most relevant deHERVs according to this analysis are commented on individually in the discussion section.

Overall, the division of RTP individuals in two clusters according to TPM analysis is in line with the fact that they represent clinically active infections and can hence be viewed as a transition step toward convalescence. In a broader view, these results might indicate the presence of a minority of HERV loci whose expression is modulated by the general activation of innate immunity, hence possibly acting as sentinels of a sort for the different infections, while another portion might show transcriptional changes specifically linked to the presence of a certain virus. Hence, to further explore this possibility, we extracted from the Gencode set ~1,100 genes based on the list provided by InnateDB (https://www.innatedb.com), a publicly available database of the genes and signaling pathways involved in the innate immune response. Aiming to assess if their dynamic modulation across the different conditions had some common traits with respect to our key HERVs, we generated a heatmap of the InnateDB genes’ variance (Fig. S6) and compared it to the heatmap of HERV expression, as obtained from the top 500 HERVs with the highest variance (Fig. 2B, reported also in Fig. S6B to facilitate the comparison). Intriguingly, the two heatmaps are perfectly comparable, showing similar behavior both for the blocks of immune genes and HERVs that show a common modulation under the different conditions (as represented by the rows’ dendrogram) and for the clustering of samples (as shown in the columns’ dendrogram).

### *De novo* reconstruction of putative transcripts from the 20 selected key deHERVs.

Finally, given that the 31 key deHERVs are modulated under all conditions, we wanted to reconstruct their potential transcripts from raw reads to assess their coding potential as well as their interaction with those of colocalized/nearby cellular genes. To this purpose, we applied the Trinity pipeline for *de novo* transcript reconstruction for the samples as divided into HC, RTP, and C conditions (Table S8, Fig. S7).

Analysis of the inferred transcriptomes confirmed a good relation between TPM values as calculated for the whole locus and the actual transcripts’ production capacity. In fact, on he one side, the deHERVs that showed low expression values based on the above-described TPM threshold (mean TPM, >2.5 under at least one condition; Table S7) were not associated with the relevant transcripts (data not shown). On the other side, for the remaining 20 key deHERVs, TPM values under the three conditions had a direct link with the presence of different isoforms of potential interest. Indeed, the 12 key deHERVs associated with potentially relevant transcripts have been characterized in terms of genic structure, coding potential, and interplay with nearby cellular genes (Table S8, Fig. S7).

As already mentioned, most of the transcripts showed isoforms specific to the HERV locus and/or in the same orientation under the condition with the highest TPM expression. For example, HERV-E locus 2776 had the highest TPM under the C condition (17.9 versus 2.0 and 4.2 in HC and RTP, respectively). Accordingly, in the transcriptome reconstructed from C samples, we found a predicted transcript (*2776_C_TRINITY_DN12_c0_g1_i2*, 3,024 bp) that overlaps the HERV locus (at *pol-env* genic portions) with the same orientation, while HC and RTP transcriptomes included only shorter and antisense transcripts (Table S8, Fig. S7). A similar situation was observed for the HERV-E locus 3949 (*3949_HC_TRINITY_DN2_c1_g2_i1, c1_g2_i4*, *g2_i8*, and *c1_g2_i9*), the HERVIP10 locus 6228 (*6228_HC_TRINITY_DN3_c1_g5_i1* and *c1_g5_i2*), the HERV-H48 locus 5410 (*4332_HC_TRINITY_DN3_c2_g1_i2* and *c2_g1_i7*), and the HERV-H locus 933 (*933_HC_TRINITY_DN1_c1_g3* and *c1_g3_i7*) (Table S8, Fig. S7). Another example of transcripts in different orientations according to the condition is HML3 locus 4332, which was highly expressed in C individuals (TPM, 66.3 versus 8.5 and 24.6 in C and RTP, respectively). Accordingly, only under this condition did we find sense transcripts: some of them were colinear to the HERV structure (*4332_HC_TRINITY_DN3_c2_g1_i2* and *c2_g1_i7*), while other isoforms included only HERV portions overlapping the long noncoding RNA (lncRNA) XR_933016, which is in the same orientation and includes only an additional portion outside the HERV (Table S8, Fig. S7). Similar transcripts were observed under the RTP condition showing an opposite orientation and hence potentially providing antisense complementation to the lncRNA (Fig. S7). The possible formation of dsRNA was observed in other instances and is a known and common mechanism of transcriptional silencing. For example, HERV-H locus 4613 is expressed in a transcript that seems primed by an upstream L1 element and overlaps in antisense orientation with exons 5 and 6 of the transcriptional regulator ZNF846 (*4613_HC_TRINITY_DN5_c0_g2_i14* and *c0_g2_i21*) (Fig. S7). Another possible interaction between HERV and gene expression is represented by readthrough mechanisms, in which the gene transcription is prosecuted forming a chimeric transcript including HERV-derived transcripts. HML2 locus 6171, which showed the highest TPM among the key deHERVs, is highly expressed in HC and RTP (TPM, 82.7 and 37.4, respectively). Under these conditions, a transcript produced by the already mentioned CR1 gene (*6171_HC_TRINITY_DN6_c0_g1_i3*) continues including 1,606 bp of the HERV (which is in the opposite orientation), possibly suggesting a readthrough mechanism (Table S8, Fig. S7). Other potential readthroughs may occur in HC and RTP for the HERV-W loci 14q32.11 and 3q25.1a and the colocalized genes GPR68 and ANKUB1, respectively (Table S8, Fig. S7). In the case of 14q32.11, the readthrough provides an antisense transcript (*14q32.11_HC_TRINITY_DN0_c1_g1_i1* and *14q32.11_RTP_TRINITY_DN4_c0_g1_i3I*) to the downstream gene DGLUCY (Homo sapiens d-glutamate cyclase, transcript variant 1), encoding a mitochondrial product. Finally, HERV-E locus 2574 was inferred to produce a transcript including a portion of the HERV and one of the downstream SATR1 satellite repeat, being colocalized with CZ1P-ASNS lncRNA, which itself is produced by naturally occurring readthrough transcription between the neighboring CCZ1P and ASNS genes (Table S8, Fig. S7).

Of note, none of the observed HERV-derived transcripts have retained protein-coding potential, often having a defective structure and presenting various internal stop codons and frameshifts induced by indels that mostly occur very prematurely in the protein sequence (Table S8).

## DISCUSSION

The intensive study of COVID-19 syndrome has revealed a wide range of clinical manifestations, most of which affect not only the primary site of infection, i.e., the respiratory tract, but show important and more severe systemic impairments. In particular, a main feature of SARS-CoV-2 infection is the outbreak of a potent hyperinflammatory reaction, which can cause multiple organ dysfunction and death or led to permanent and severe sequelae in the so-called postacute long-COVID syndrome (3, 28).

In this context, HERV transcriptional activation, as observed in different infectious and autoimmune diseases as well as in tumorigenesis (4, 5, 8), may sustain and amplify the inflammatory activation exerted by COVID-19, having the ability to be sensed as PAMPs by cellular PRRs and evoke an innate immune response. Such an immune trigger by HERV expression is now well characterized, given that the same immunogenic action is currently being exploited for innovative immunotherapies (5, 29–31).

Despite its possible relevance to COVID-19 symptoms and outcome, to date, a very limited number of studies has been dedicated to HERV modulation by SARS-CoV-2 infection. In addition, these few available studies are poorly comparable with the present analysis. Kitsou et al. investigated HERV group expression in the RNA-seq profiles from the bronchoalveolar lavage (BAL) fluid of 7 COVID-19 patients and 3 HC, reporting the upregulation of different HERV groups in the former (22). Indeed, a similar analysis in the PBMCs (i.e., the same biological sample considered here) from 3 COVID-19 patients and 3 HC showed an opposite panorama, with some deregulated HERV groups (HERV-I, HERV-FRD) and no significant difference for the others (22), being more in line with our findings, even if based on the expression of whole HERV groups and not on the expression analysis of their individual members. An exception was the HERV-L group, reported to be downregulated in the study, but showing 5 upregulated deHERVs out of 7 based on our analysis (22). A second study was focused only on the HERV-W group and hypothesizes that a HERV-W Env protein is expressed on the leukocytes of COVID-19 patients, correlating with inflammation and pneumonia severity (23). Our results included a total of 13 deHERV-W loci, which were downregulated in RTP and C patients except for 1 sequence that was upregulated under both conditions and another that was upregulated in RTP patients only. The other 4 deHERV-W loci specific to the RTP condition were all downregulated compared to HC, while C individuals showed 28 additional deHERV-W loci exclusive for this stage, of which 25 were upregulated compared to HC. Further comparisons are not possible, given that the genomic origin (or origins) of the observed HERV-W Env protein(s) is still unknown (23). Finally, a third study analyzed by reverse transcriptase PCR (RT-PCR) the generic expression of HERV-H, HERV-K(HML2), and HERV-W *pol* genes as well as of the HERV Env proteins syncytin-1 and -2 in a cohort including HC and children with either mild/moderate symptoms, severe symptoms, or multisystem inflammatory syndrome (MIS), evaluating also the IFN response (24). The authors found that children with mild manifestations had increased mRNA levels of HERV genes compared to those with severe symptoms (significantly for all but syncytin-1 and -2) or MIS (significantly for all, but borderline for syncytin-1) and that such expression is correlated with the levels of TRIM28 or SETDB1 (24). Also, in this case, it is difficult to compare these results with our high-throughput analysis, given that RNA was extracted from whole blood and analyzed with a different technique and, especially, because the individual HERV-H, HERV-W, and HERV-K(HML2) members that were amplified by the *pol* primers used in each group are not known. Similarly, also, the actual specificity of syncytin-1 and -2 primers should be carefully assessed to avoid cross-amplification of different HERV loci. Besides these limitations, based on our results, no modulation of syncytin-1 or syncytin-2 loci (HERV-W 7q21.2 and HERV-FRD 6p24.1, respectively) was observed, at least in the PBMCs of adult SARS-CoV-2-exposed individuals.

Hence, most of the previously available studies had a resolution limited to the generic HERV group level, meaning that no information is available for the modulation of the individual HERV loci (which, for example, are at least 213 in number in the case of the HERV-W group) but is only available for the overall expression of the different HERV groups. This approach is of course less informative, given that the actual contributors of the observed modulation in terms of HERV sequences remains unknown among the different conditions. In addition, most of these studies were focused on cells that originated from biological sites directly affected by the infection, such as respiratory cells from bronchoalveolar lavage fluid or rhino-pharyngeal swabs.

Indeed, in the present study, we aimed to investigate the modulation of the HERV transcriptome in PBMCs. It is important to underline that, on the one hand, these cells are not infected by SARS-CoV-2, and hence can not account for variations directly associated to viral infection and presence. On the other hand, PBMCs represent cellular immune effectors and are hence highly relevant to antiviral responses, having a central role in the plethora of inflammatory and immune events that characterize COVID-19 pathogenesis and sequelae. For this reason, we thought to assess the transcriptional variations in PBMCs of individuals exposed to SARS-CoV-2, after either viral clearance (convalescence) or the recurrence of the infection (retesting positive).

Sample-to-sample distance and PCA analysis based on the expression of ~3,300 individual HERV integrations confirmed that SARS-CoV-2 infection has a major impact on the HERV transcriptome in PBMCs, which showed a different pattern of modulation both in the exposed individuals (RTP+C) with respect to HC and, especially, according to the presence of an active infection (RTP) or a past exposure to the virus (C), being hence able to divide the samples according to their COVID status (Fig. 1A). In line with this, the past or current presence of SARS-CoV-2 infection accounts overall for the 58% of the total variance observed in HERV expression: based on HERV transcriptional variation, we were able to divide HC from patients that were exposed to SARS-CoV-2 infection, regardless their current clinical status (PC1, 45%), but also patients with an active infection from healthy individuals and recovered patients (PC2, 13%) (Fig. 1B).

This confirms, on the one hand, that SARS-CoV-2 infection is associated with a long-term effect on the host, which is still evident after viral clearance and in a cellular population being not directly infected by the virus, and on the other hand, that such modulation has different and specific signatures in actively infected patients and individuals recovered from the infection when considering HERV expression.

It is noteworthy that the same analyses performed on the expression of cellular genes did not have a comparable resolution for COVID19 exposure. In fact, while the sample-to-sample distance plot based on the global cellular gene expression showed a similar division according to COVID-19 status, the PC analysis conducted on the expression data from cellular genes did not distinguish the different conditions, suggesting that factors other than the exposure to the infection have a major impact on their transcriptional variation (Fig. S1). We then hypothesized that the individuals could have an intrinsic interindividual variability in their response to the infections, given that most of the infected patients (RTP) were clustered with HC by the PC1 (54%). To gain further insights on this, we restricted the analysis to a subset of 44 key innate immune genes that have been reported as a specific signature of induced cytokine response (26), which is a main characteristic of COVID-19 hyperinflammatory pathogenesis (3). Accordingly, sample-to-sample distance and PC analyses performed with these 44 immune genes showed their clear division into two major groups with opposite immune signatures: one including two clusters formed by all C patients and 6 RTP patients, and the other including the rest of the RTP patients (4) and all the HC (Fig. S2). It is hence reasonable that this second subset of RTP patients is formed by low-responder individuals, thus showing a transcriptional pattern like that of the HC. In this case, the PC1 (65% of the variance) is represented by the different signature of the induced cytokine response. Another possibility could be that these RTP patients represent false positives, but this is unlikely since the analyses performed with the whole set of cellular genes, even if less specific for this immune signature, already grouped RTP patients into two subclusters that were clearly divided from the other two categories (Fig. S1A). Also, the observed differences cannot be linked to an imbalance in the immune cell subset in RTP patients, since they were shown to have a stable number of immune cells compared to the others (25). It is, instead, possible to speculate that these RTP individuals, due to their status of immune low responders, could have had only a partial viral clearance after the primary infection, showing a rebound of the same virus after an apparent negative period. This is in line with the fact that they showed weakly activated T cells in the original study, which may contribute to the obstruction of SARS-CoV-2 clearance (25) and would also explain their slight division from the other RTP patients, representing in its place true reinfections. Notably, this possibility is also in line with the distribution of samples based on the HERV PC1, in which 2 of the RTP low responders are in the half of the plot with HC, and a third are near the PC1 axis (Fig. 1B). A similar influence on the HERV transcriptome has already been reported after the *in vivo* administration of lipopolysaccharides, mimicking a bacterial infection (10). In this case, however, the impact of the immune status on HERV variance seems to be a minor component, also given that none of the deHERVs were colocalized with any of the 44 immune genes.

The generation of heatmaps based on the selection of the 500 HERV sequences showing either the highest mean or variance of expression (Fig. 2A and B) further confirmed that the individual HERV loci are grouped in blocks based on their specific modulation under the different conditions, and these transcriptional pattens allow clear divisions of HC, C individuals, and RTP patients. Moreover, as already suggested by the PCA analysis, RTP patients represent the most heterogeneous group in terms of HERV transcriptional variation, being sometimes more similar to the HC transcriptional pattern yet still separated from this well-defined cluster (Fig. 2B). The fact that a similar behavior was shown with the same selection on cellular genes likely indicates that the indirect impact of SARS-CoV-2 infection on the PBMC transcriptome is a major determinant of transcriptional modulation, influencing the expression of both canonical genes and endo-retroviral loci.

Having the evidence of a global modulation of HERV by SARS-CoV-2 exposure, we then moved to the locus level, identifying the individual HERV integrations that were modulated under the different conditions. To this purpose, we performed multiple differential expression analyses: on (i) the overall data set, comparing exposed individuals to HC to identify deHERVs modulated regardless of status of RTP or C, as well as on specific subsets, including (ii) C and (iii) RTP individuals individually compared to HC, to assess the specific modulation by past or present exposure to the infection, respectively, and (iv) C and RTP individuals (without HC) to assess the HERVs significantly modulated between the two clinical stages.

The overall analysis (C plus RTP versus HC) identified 282 deHERVs; of these, 72 were upregulated (*P*-adj values of 0.01 to 6.22^−14^) and 210 were downregulated (*P*-adj values of 0.01 to 1,05^−40^) in individuals exposed to SARS-CoV-2 infection (Fig. 3A, Table S2).

To gain more insights on the basis of deHERV modulation in SARS-CoV-2-exposed individuals, we assessed their colocalization with cellular genes, which can themselves be modulated by the infection and hence influence HERV expression. Among the 282 deHERVs found in the study, 170 (~60%) were colocalized with a total of 205 cellular genes, only a minority of which were modulated by the infection (50 genes, 24%) (Fig. 4A, Table S3). In particular, we observed a concordant modulation between the deHERV and the colocalized de-gene, except for the deHERV locus 2328 (HERV-H), which was downregulated in RTP and C individuals, while the surrounding gene RNF217-AS1 was upregulated under the same conditions. The fact that the deHERVs showed a concomitant modulation with the colocalized gene can be somewhat expected by the fact that a HERV sequence inserted within a gene is likely influenced by the latter’s transcriptional activity. RNF217-AS1 is among the four lncRNAs reported to have important effects on the survival prognosis of esophageal squamous cell carcinoma (ESCC), being significantly upregulated in these patients compared to HC. It may be possible that a similar upregulation can be linked to the important immune activation by COVID-19 infection. Another interesting aspect highlighted by the analysis is that most of the deHERVs colocalized with de-genes show a concomitant negative modulation in the individuals exposed to SARS-CoV-2 (Table S4). This is somewhat unexpected, given that we thought that most of the deHERVs colocalized with modulated cellular genes would have been activated, likely because those genes were involved in inflammatory and immune responses. In contrast, only 8 among the 48 deHERVs with concordant modulation to the colocalized de-genes were upregulated in RTP and C patients (Fig. 4A, Table S4). These included 2 noncoding genes, 4 genes involved in the immune response against infectious agents (COLEC12, TNFRSF21, PKHD1, PRKAR2B), and 2 genes with different activity (FLVCR2, of retroviral origin, and PRRG1) (Table S4). The remaining 41 downregulated de-genes comprised 28 nonprotein coding elements and 13 protein-coding sequences with different functions, also including 2 retroviral genes associated with the encompassing HERV locus (Table S4). In addition to a HERV-48 locus in chromosome 21, producing a noncoding RNA (ERVH48-1), a downregulated HERV gene potentially encoding an Env protein was found at chromosome 7 (ERV3-1, HERV-3 locus 2521). Besides its own *env* gene, this HERV is colocalized also with ZNF117, a cellular gene containing multiple zinc finger motifs, which was also downregulated. This is in line with the fact that readthrough transcription has been reported between this gene and the upstream ERV3-1 locus (14).

Concerning the HERV groups modulated by exposure to SARS-CoV-2, among the 282 deHERVs from the overall analysis, the majority belong to class I HERV groups (80%), being HERV-H elements in half of the cases (Fig. 3B). This result was expected, given that Class I HERVs are the most abundant, and HERV-H is the group with the highest number of members (27, 32). Similarly, within the class II HERV-K groups (16% of deHERVs), HML2 was the most represented, possibly also due to its relatively recent activity that led to recent, human-specific integrations (33), followed by HML3, HML5, HML1, and HML6. The remaining 4% of deHERVs were divided between the class III HERV-L group (7/282) and unclassified elements (4/282) based on our recent comprehensive classification (27). Except for the HERV-L group, including 5/7 upregulated deHERVs (71%), all the other HERV groups showed a prevalent downregulation of the respective deHERV members in individuals exposed to SARS-CoV-2, with only 0 to 30% of deHERVs being upregulated (except for HERV-I, including 1 upregulated and 1 downregulated member). Clearly, this result is likely more representative of the RTP status, given that RTP individuals accounted for 10/16 of SARS-CoV-2-exposed individuals.

In the light of this and given that no previous studies assessed HERV differential expression among different COVID-19 clinical stages, we also characterized the specific modulation of HERV transcriptome under each condition (RTP and C individually compared to HC) and between past and current SARS-CoV-2 exposure (RTP versus C).

All the 282 deHERVs identified in the global analysis (RTP and C versus HC) showed a concordant modulation in the RTP versus HC subcomparison (Table S5 and Fig. 4B), as expected from the slight imbalance between the numbers of the two conditions. However, the 72% of these deHERV loci were shared in the C versus HC subcomparison as well (202/282), confirming a common transcriptional signature acting on specific HERV loci in the presence (either past or current) of SARS-CoV-2 infection. Furthermore, among the 202 deHERVs shared between the RTP and C subcomparison, 99% shared a concordant modulation compared to HC (70 upregulated deHERVs and 130 downregulated deHERVs), with only 2 discordant deHERVs that were upregulated in C and downregulated in RTP.

Intriguingly, the C versus HC subcomparison revealed that, besides the persistence the above deHERVs shared with RTP-infected patients, the past exposure to COVID-19 triggers a delayed and wider transcriptional modulation of HERV elements in C individuals, with additional HERV loci (369) that become modulated only during convalescence, while only a minority of elements (80) return instead to the original, basal condition in after the transcriptional changes observed in the presence of the active infection (Table S5, Fig. 4B). Moreover, contrary to the modulated HERVs shared with the RTP condition, the deHERV loci specific to C status were mostly upregulated (305/369, 83%). In our hypothesis of a close connection between immune activation and HERV modulation, this result is in line with the observation by Wang and coworkers, reporting that negative regulators of immune system and lymphocyte activation signaling were enriched in PBMCs from RTP patients (25). The same scenario was also confirmed by our GO analysis of upregulated de-genes colocalized with comodulated de-HERVs in RTP individuals compared to HC and C individuals in the subcomparison analyses (Fig. S4, Table S6). In fact, even if the immune genes involved were different, the two most significant GO processes were associated with the impairment of cellular immune effectors in both RTP versus HC (*P* = 0.0020, TNFRSF21 gene) and RTP versus C (*P* = 0.0057, CR1 gene) (Fig. S4, Table S6).

Excluding HC, the differential expression analysis between the RTP and C conditions identified 164 deHERVs: of these, 51 (31%) were also modulated in the overall comparison (C and RTP versus HC), including 31 deHERVs shared by both the RTP and C subsets and 20 other specifically modulated in RTP individuals (Fig. 4B). The remaining 119 were instead upregulated in the C state, further confirming a specific HERV upregulation associated with this stage.

Of note, we identified 31 key deHERVs, whose expression was significantly modulated in all COVID-19 stages (C versus HC, RTP versus HC, RTP versus C) (Table 1, Fig. 5). The fact that these loci belonged overall to 14 HERV groups (Fig. 5A) confirms that HERV modulation occurs at the individual locus level, requiring high-throughput transcriptional analyses of each individual group member instead of generic, whole-group expression studies. The latter have been often performed using primers and probes with uncertain coverage and selectivity, creating general confusion and a lot of unreliable tentative associations that somehow undermined the field’s credibility. Most of these key deHERVs showed a concordant modulation in SARS-CoV-2-exposed patients, being either downregulated (19) or upregulated (10) in both C and RTP compared to HC (Table 1). TPM calculation provided more insights into their transcriptional modulation, revealing three clusters (on the *y* axis): one with deHERVs with higher expression in C (cluster 1, *n* = 10), a second with higher expression in HC (cluster 2, *n* = 16), and a third with higher expression common to C and HC (cluster 3, *n* = 5) (Fig. 5B). In the first two clusters RTP showed an intermediate TPM, which is in line with the fact that they represent clinically active infections and can hence be viewed as a transition step toward convalescence. To our knowledge, this is the first set of deHERVs whose expression is dynamically modulated across COVID-19 clinical stages.

Based on this analysis, we also confirmed that some of the RTP patients are likely low responders for the key innate immunity genes analyzed previously (Fig. S2), forming 3 clusters based on deHERV TPMs (Fig. 5B). While the second and third RTP clusters were related to C, according to their common exposure to SARS-CoV-2, the first was related to the HC group, including 3 out of 4 supposed low responders that showed a transcriptional behavior similar to that of HC in the key innate immunity gene analysis (marked with a asterisks in Fig. 5B). Interestingly, such a common modulation was not evident in the expression of all the 31 deHERVs but seemed to influence only a subset of 9 deHERV loci (Fig. 5B). The other RTP predicted to be a low responder was instead included in the third group but also showed deHER versus TPM more similar to that of the first RTP cluster. These results might indicate that, in the context of viral infections, a portion of de HERV loci are modulated by the activation of innate immunity, possibly acting as sentinels of a sort for any kind of infection, while another portion show transcriptional changes specifically linked to the presence of a certain virus. This scenario is further supported by the fact that the heatmap based on the variance of around 1,100 cellular genes involved in innate immune responses shows a clustering of samples and an overall transcriptional pattern that is comparable to those of the top 500 HERVs, also in terms of dynamic variation across conditions (Fig. S5).

We then selected and analyzed in detail the 20 deHERVs showing a TPM of >2.5 under at least one condition, distinguishing them based on the colocalization with cellular de-genes (*n* = 7, Fig. 6) or lack of colocalization (*n* = 13, Fig. S5). The highest TPM values were found for HML2 locus 6171 at chromosome 1 (mean TPM of 82.7 in HC, 37.4 in RTP, and 9.9 in C): this HML2 was inserted in antisense orientation within the CR1 gene, encoding a protein that mediates cellular binding of particles and immune complexes that have activated the complement (Fig. S5). Of note, even if this gene was not differentially expressed in the main analysis (RTP plus C versus HC) (Table S3, Fig. S5), CR1 and the colocalized HERVs were both upregulated in the PBMCs of RTP compared to C individuals, and CR1 modulation was predicted to affect immunoglobulin production and B cell differentiation based on the above-mentioned GO analysis (Fig. S4, Table S6). The *de novo* transcript reconstruction in HC and RTP individuals showed a possible readthrough between this gene and the HERV that, being in the opposite orientation, can eventually provide a silencing mechanism under these conditions according to TPM values (Table S8, Fig. S7). The second and third deHERVs based on TPM values belonged to class I, being, respectively, the HML8 locus 15q15.1 and the HML3 locus 4332. Like the first one (HML2 locus 6171), both were upregulated in HC and showed the lowest expression in C individuals, and in addition, they were colocalized with two non-protein-coding de-genes (Fig. 6, Table S3). In particular, the HML8 locus 15q15.1 (mean TPM of 69.2 in HC, 33.5 in RTP, and 8.6 in C) is integrated within RAD51-AS1, producing an uncharacterized antisense RNA to the RAD51 recombinase gene that is, in turn, involved in homologous recombination and repair of DNA, while the HML3 locus 4332 (mean TPM of 66.3 in HC, 24.6 in RTP, and 8.5 in C) is integrated within AC108134.3/XR_933016, producing an uncharacterized long noncoding RNA (lncRNA). The analysis of these two deHERVs was done also considering the expression of the colocalized genes and showed that the latter have a modulation concordant with the colocalized HERV gene but with lower TPM values (Fig. 6). If, on the one hand, it is thus unlikely that the genes’ modulation has an impact on the deHERVs expression, on the other hand, the fact that both these uncharacterized genes have annotated exons within the respective HERV sequences leaves open the possibility that HERV modulation can, in turn, influence their transcription. Accordingly, transcript reconstruction suggested a possible readthrough between XR_933016 exons, with the HERV sequence present in some isoforms, and a similar but antisense transcript in RTP may led to the formation of dsRNA (Table S8, Fig. S7). A similar concordant modulation, with a lower gene expression, was observed for the HERV-E loci 2574 in chromosome 7 (mean TPM of 5.4 in HC, 1.9 in RTP, and 0.4 in C), being integrated within introns of two uncharacterized lncRNAs. HERV-E locus 2574 is colocalized with CZ1P-ASNS lncRNA, produced by naturally occurring readthrough transcription between the neighboring CCZ1P and ASNS genes, being possibly derived from a similar mechanism (Table S8). Two of the remaining three deHERVs integrated within de-genes were found within noncoding sequences as well, showing concordant modulation having slightly lower TPM values in this case: the HERV-H locus 3052 in chromosome 9 (mean TPM of 3.1 in HC, 0.4 in RTP, and 1.0 in C) was colocalized with the uncharacterized lncRNA gene AL355377.4, and the HERV-H48 locus 5410 in chromosome X (mean TPM of 5.1 in HC, 0.4 in RTP, and 0.05 in C) was colocalized with the uncharacterized lncRNA gene Z83745.1 (Fig. 6). In both cases some exons were annotated and overlapped the HERV structure, suggesting that these transcripts might originate from these two elements. Finally, the HML3 locus 2135 in chromosome 6 was one of the two deHERVs upregulated in SARS-CoV-2-exposed individuals—with the highest expression under the C condition (mean TPM of 0.1 in HC, 0.5 in RTP, and 8.9 in C)—as well as the only one among the deHERVs colocalized with de-genes (Fig. 6). Of note, the colocalized gene showed an overlapping transcriptional behavior and was the already mentioned TNFRSF21, which plays a role in T helper cell activation, inflammation, and immune regulation in the presence of infectious agents. Like CR1, this gene and the colocalized HERVs were both upregulated in the PBMCs of C and RTP individuals compared to HC, and such TNFRSF21 modulation was linked to the negative regulation of interleukin-13 and -5 production based on the previously mentioned GO analysis (Fig. S4, Table S6). It is hence likely that the HERV modulation can be influenced by the activation of this gene in SARS-CoV-2 infection and along COVID-19 convalescence, accounting for its specific upregulation in RTP and C individuals.

Among the deHERVs that are not colocalized with de-genes (*n* = 13), beside the above-mentioned HML2 locus 6171 that was the one with the highest TPM, some other elements showed a relevant level of expression (Fig. S5). Among these, only the HERV-E locus 2776 in chromosome 8 was upregulated in SARS-CoV-2-exposed individuals, also in this case with the highest expression under the C condition (mean TPM of 2.0 in HC, 4.2 in RTP, and 17.9 in C) (Fig. S5B). Accordingly, a specific predicted transcript including the HERV and showing the same orientation was inferred under the C condition only (Table S8, Fig. S7). This HERV is integrated within an intron of the GPAT4 gene (glycerol-3-phosphate acyltransferase 4) that was not differentially expressed in the overall analysis (Table S3). However, the GPAT4 TPM shows the same behavior as the deHERV under the different conditions, suggesting that the gene can have some impact on the HERV upregulation (data not shown). Most of the other deHERVs were downregulated in SARS-CoV-2-exposed individuals, further confirming that the presence of infectious and immunogenic conditions is not necessarily associated with a general activation of HERV expression. Still, in this case the dynamic gradient of TPM was evident, with the highest expression being in HC, followed by RTP, and the lowest under the C condition in most cases (Fig. S5A).

In general, besides the possible occurrence of readthrough mechanisms and formation of dsRNA, the *de novo* transcript reconstruction for the key deHERVs that were dynamically modulated under all conditions also showed that these elements are far from being the most preserved, often showing a defective structure with several indels that generate frameshifts in the coding region, as well as nucleotide substitution that often led to the introduction of premature stop codons. Even if this prediction has clear limitations, in the scenario indicating a possible role of these HERVs in the dynamic transition, such a defective structure may be the result of a specific selection, leading to the production of specific transcripts under certain conditions of immune modulation and preventing the production of potentially harmful retroviral proteins. Of course, further studies are needed to clarify this complex interplay between HERVs and cellular immunity, which started since mammalian evolution with a pivotal role in the shaping of antiviral responses (7) but is still far from being fully understood. In particular, since a limit of the present study is the lack of primary infections, it will be important to complete the overview presented here by characterizing the transcriptional profile of acute SARS-CoV-2 infections and compare it to the one observed in patients who retest positive. Also, it will be interesting to test the identified key deHERVs in cellular systems to assess their possible impact on immune gene activation and *vice versa*.

**Conclusions.** Overall, our results provide some interesting and new insights about HERV modulation by SARS-CoV-2 and, especially, its dynamic evolution along COVID-19 clinical stages.

On the one hand, the fact that a majority of deHERV loci as identified in RTP individuals are also shared with the C condition (202/282), showing a concordant modulation in 99% of cases, reveals that SARS-CoV-2 exposure has a prolonged influence on a subset of HERV loci, which show the same stable modulation even after the clearance of the infection. It is worth noting that the majority of these common deHERVs are downregulated. In fact, the common idea is that the presence of infectious agents can led to a general upregulation of HERV expression, as reported, for instance, for HIV (12–15), HCV (16), influenza (17), herpesviruses (18–21), and other viral infections (5, 34, 35). Based on these observations, it has been proposed that HERV elements have been exploited by the innate immunity machinery to improve antiviral responses against current infectious agents (5, 36). In our case, considering SARS-CoV-2 infection, it seems that such an activation occurs later, i.e., during convalescence instead of in actively (re)infected individuals. Whether this is linked to the inefficient antiviral response to SARS-CoV-2 is still to be clarified. Also, according to the hypothesis of a role of HERV modulation toward the trigger of antiviral responses, it is not mandatory that the infection has an activating action on HERV loci, because the significant reduction of certain HERV transcripts can provide biological signals (e.g., if the transcript is involved in interference and/or silencing of immune effectors).

On the other hand, the preponderant additional deHERV activation specific to the C stage is in line with the growing evidence that COVID-19 is linked to the persistence (or even the onset) of severe and systemic sequelae after SARS-CoV-2 infection has been cleared, leading to postacute long-COVID syndrome (28). In particular, the evidence of a dynamic and progressive transcriptional activation of HERV subsets during convalescence can indicate a role of HERV expression (among the other factors) in the major inflammatory signature of long-COVID syndrome and deserves further investigations considering patients with primary infection as well.

In this context, however, it is more likely that, instead of a general, unspecific upregulation of whole groups of endogenous elements by viral infections, only a minority of HERV sequences are modulated in the presence of infectious agents, acting as immune sentinels for antiviral responses. For example, a subset of specific mammalian ERVs are known for their central role in the shaping and evolution of the IFN transcriptional network, a crucial weapon for innate immune responses (7). In this complex interplay, it is probable that factors other than those involving the HERV group have a major impact on the modulated HERVs, including the proximity to immune genes, requiring investigation at the locus level rather than general analysis of a whole HERV group modulation (37, 38).

## MATERIALS AND METHODS

### Collection of public transcriptomic profiles.

The RNA-seq raw data analyzed in the present study were generated by Wang and coworkers (25) and retrieved from the Gene Expression Omnibus (GEO) repository (accession number GSE166253). In particular, this data set was chosen due to its technical parameters (paired-end reads of 150 + 150 bp, around 40 million per sample), which were suitable for the univocal detection of reads mapping to repetitive multicopy elements. As previously mentioned, the data set included a total of 26 RNA-seq profiles from either convalescent COVID-19 patients (C, *n* = 6), patients that retested positive to SARS-CoV-2 after convalescence (RTP, *n* = 10) and healthy controls (HC, *n* = 10). The population showed a mean and median age of 65 years and included 12 females and 14 males (Table S1). Raw RNA-seq data were downloaded via ftp from the European Nucleotide Archive (ENA; www.ebi.ac.uk/ena/) and subjected to quality control with the software FastQC (39) prior to subsequent analyses. A quality check confirmed that all FASTQ files included reads of 150 bp in length, without uncalled bases and with satisfactory quality scores (Table S1).

### Bioinformatic pipeline for HERV expression analysis.

For each sample, the corresponding paired FASTQ file was first aligned to the reference human genome sequence (GRCh38/hg38) using the STAR aligner version 2.5.2 (40). Then, the Python library HTSeq-count (41) was used to quantify the reads mapping to each of the ~3,300 individual HERV loci included in our HERV data set (HERVdb), relying on their univocal genomic coordinates (27, 42–47). The same framework was used to count the reads mapping to all the human genes included in the Gencode data set version 34 (48). Raw counts were analyzed with RStudio software version 1.4.1106 (49) to estimate the relative abundance of reads and to perform differential expression analyses of HERV loci and cellular genes among the different conditions (C and RTP versus HC, C versus HC, RTP versus HC, C versus RTP). In particularly, the relative abundance of reads was calculated as transcripts per million kb (TPM) expression values, while differential expression analyses were performed from raw counts with the DESeq2 package (50), setting as the statistical threshold a Benjamini-Hochberg adjusted *P* value (*P*-adj) of ≤0.01 and an absolute log_2_ fold change (log2FC) of ≥1.

### Characterization of deHERV loci.

The individual HERV loci found to be differentially expressed (deHERV) under the different conditions were classified and aligned to the corresponding reference sequence from the Dfam database of repetitive elements (51) to infer their genic structure and evaluate their completeness and coding potential. The genomic context of integration of deHERV loci was characterized by intersecting their univocal coordinates with those of the complete set of human genes (Gencode34). Gene Ontology analyses of upregulated de-genes colocalized with comodulated deHERVs were performed with the ModEnrichr suite and GO Biological Process 2021 annotations (52). For the 31 key de-HERVs modulated under all conditions, the corresponding means of expression and those of eventually colocalized genes (expressed as TPM values) were statistically compared between C and RTP individuals using a two-sample *t* test.

### *De novo* reconstruction of transcripts.

Raw RNA-seq data of the three different sets of individuals were used for the *de novo* reconstruction of the PBMC transcriptomes for HC, C, and RTP conditions using Trinity software (53). Reconstructed transcripts were mapped back to the human genome reference sequence with GMAP mRNA aligner (54) and visualized in the context of the human genome with Integrative Genomics Viewer (IGV) software (55).
