# Supplementary material for: Human Endogenous Retrovirus (HERV) Transcriptome Is Dynamically Modulated during SARS-CoV-2 Infection and Allows Discrimination of COVID-19 Clinical Stages
Source: Microbiol Spectr. 2023 Jan 5;11(1):e02516-22. doi: 10.1128/spectrum.02516-22 (PMC9927238; doi:10.1128/spectrum.02516-22)
Supplement: Supplemental file 1 — Supplemental material. Download spectrum.02516-22-s0001.pdf, PDF file, 7.2 MB [file spectrum.02516-22-s0001.pdf]

A

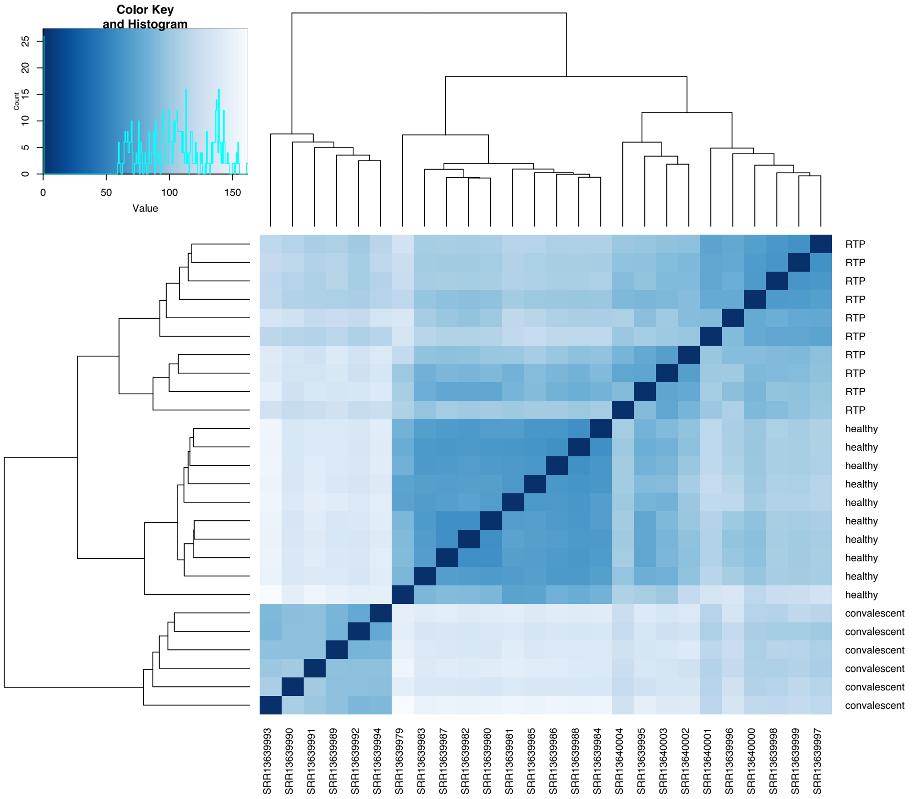

B

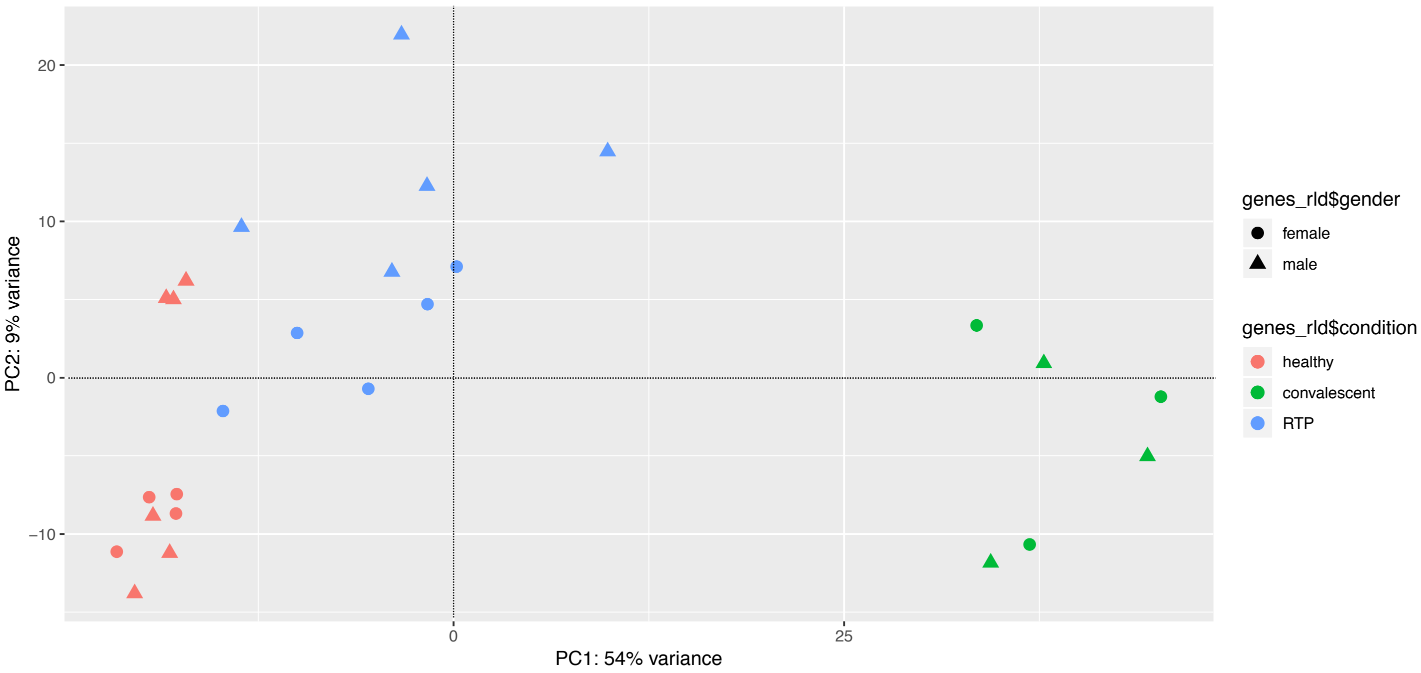

Supplementary Figure S1. Sample to sample distance (A) and PCA plot (B) of ~60700 cellular genes

(A) Heatmaps of the overall similarity between samples: correlation distance measure has been used in clustering columns based on the rlog-normalized expression data of the Gencode v34 set of ~60700 cellular genes. Distance values are blue scaled, as represented in the color key and histogram legends.

(B) Principal Component Analysis on rlog-normalized cellular genes' expression data. Samples are annotated by condition: red, healthy controls; green, convalescent after recover from SARS-CoV-2 infection; blue, re-testing positive after convalescence.

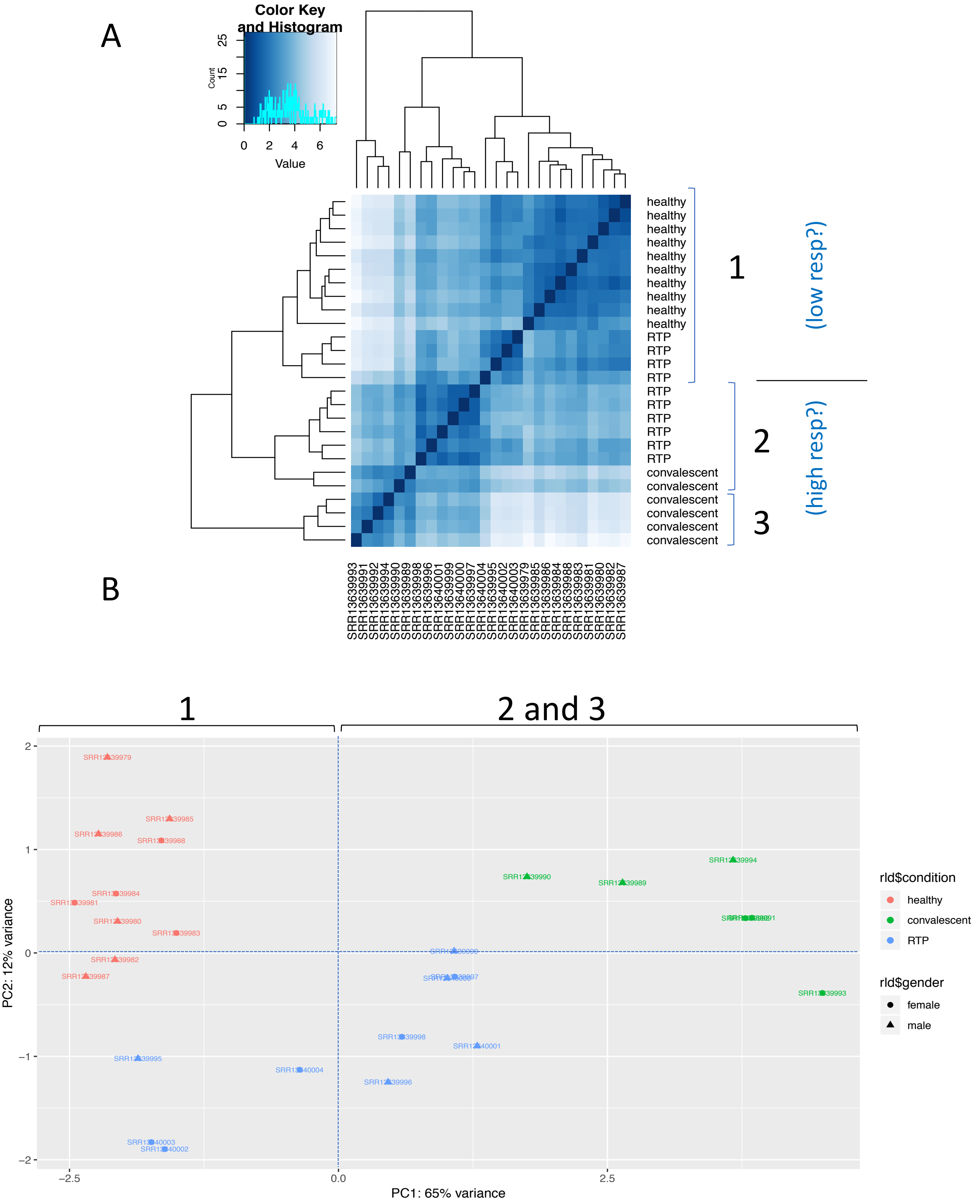

Supplementary Figure S2. Analysis of a subset of 44 cellular genes able to deconvolute complex immune responses

(A)Sample to sample distance. The distance values are blue scaled, as represented in the color key and histogram legends. High and low RTP responders showed different responses to inflammation.

(B) Principal Component Analysis as performed on rlog-normalized expression data of the 44 immune genes. Samples are annotated by condition: red, healthy controls; green, convalescent after recover from SARS-CoV-2 infection; blue, re-testing positive after convalescence.

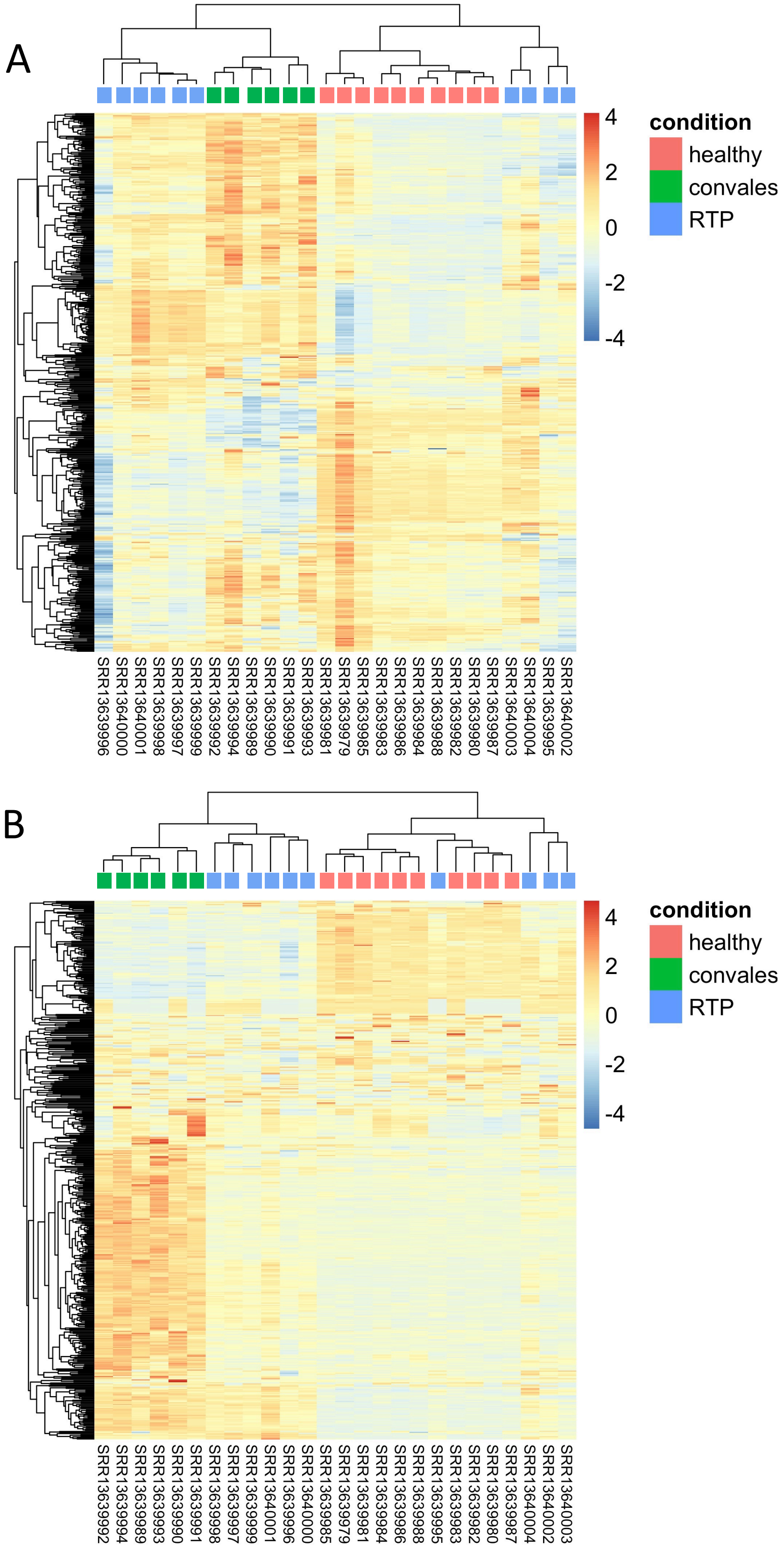

Supplementary Figure S3. Heatmaps based on the top 500 cellular genes as sorted by the highest mean (A) or variance (B) of expression.

Hierarchical clustering of the top 500 cellular genes with the highest average (A) or variance (B) of rlog-normalized counts. The top 500 genes are in rows, and the samples are in columns. rlog-normalized counts are color-scaled from blue (minimum) to red (maximum). Correlation distance measure has been used in clustering columns. Samples are annotated by condition: red, healthy controls; green, convalescent after recover from SARS-CoV-2 infection; blue, re-testing positive after convalescence.

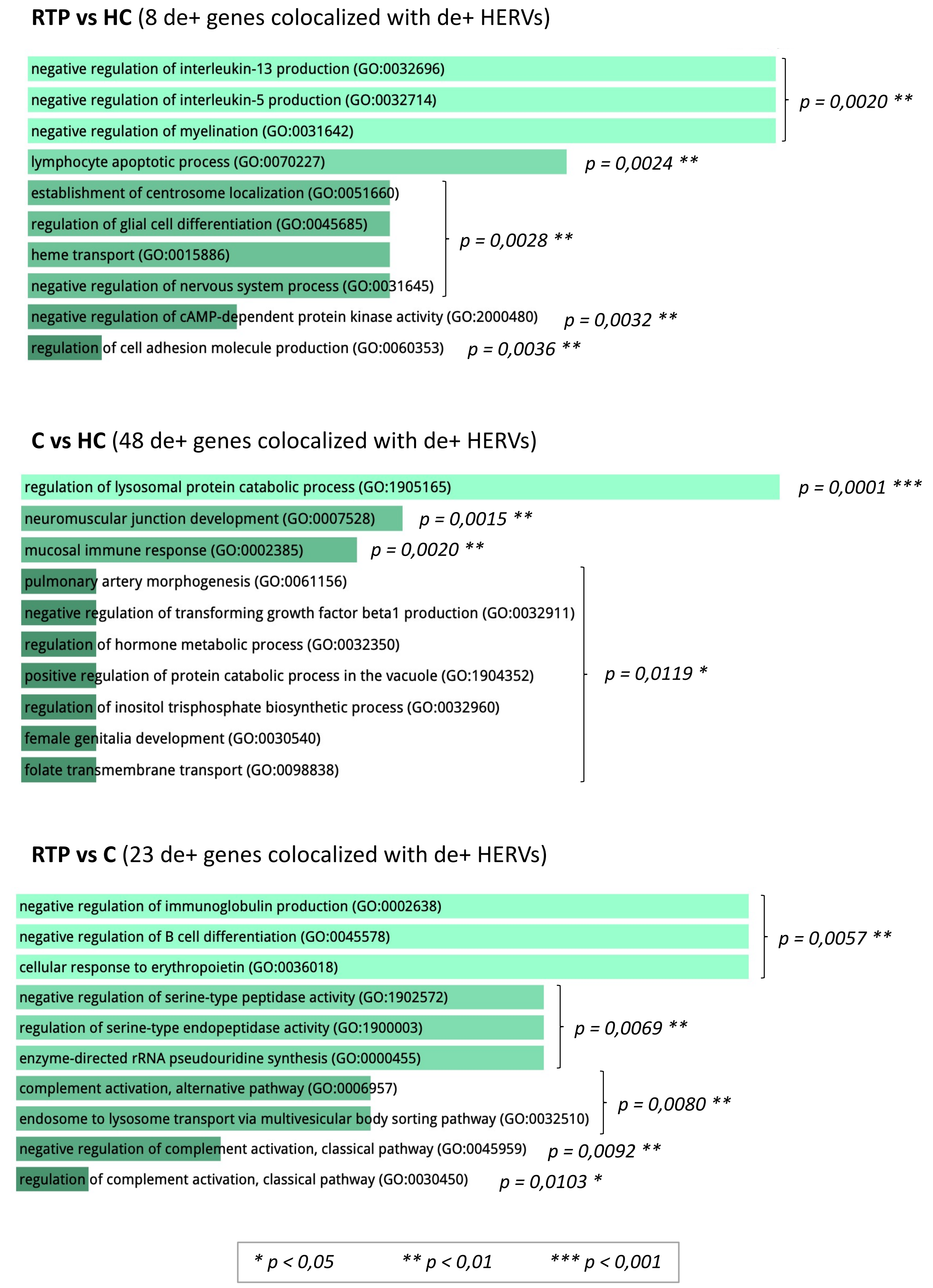

Supplementary Figure S4. Gene Ontology analysis of de+ genes colocalized with de+ HERVs in the three subcomparisons

GO analysis was performed considering a total of 8, 48, and 23 de-genes that were upregulated along with the colocalized deHERVs in RTP vs HC, C vs HC, and RTP vs C subcomparisons, respectively.

A

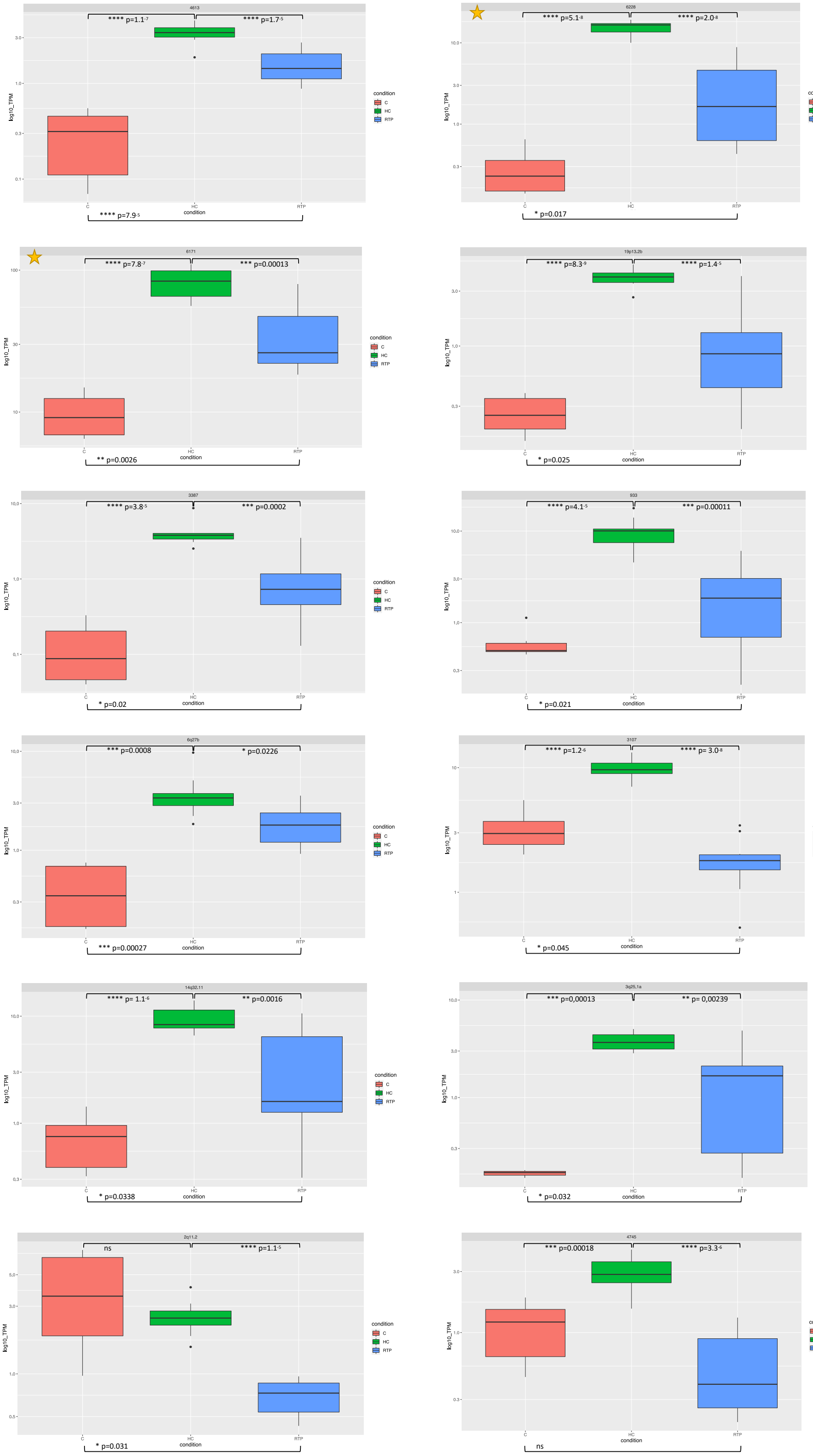

B

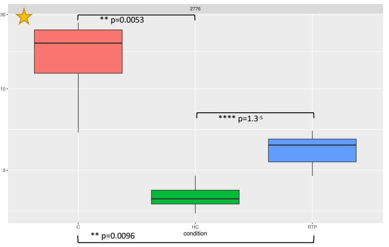

Supplementary Figure S5. Boxplot of expression levels for the key deHERVs modulated in all conditions that are not colocalized with de-genes

The expression levels (as Transcripts Per Million kilobases values, TPM) of the 14 out of 31 key deHERVs that i) are not co-localized with differentially expressed genes and ii) show TPM >2,5 in at least one condition (14/31) were plotted in the different conditions according to their magnitude of modulation: (A) deHERV down-regulated in SARS-CoV-2 exposed individuals (12/14), and (B) deHERV up-regulated in SARS-CoV-2 exposed individuals (2/14). Plots marked with a star are correspond to the deHERVs with the highest expression (log10TPM >10 for at least one condition). Statistics is based on t-test.

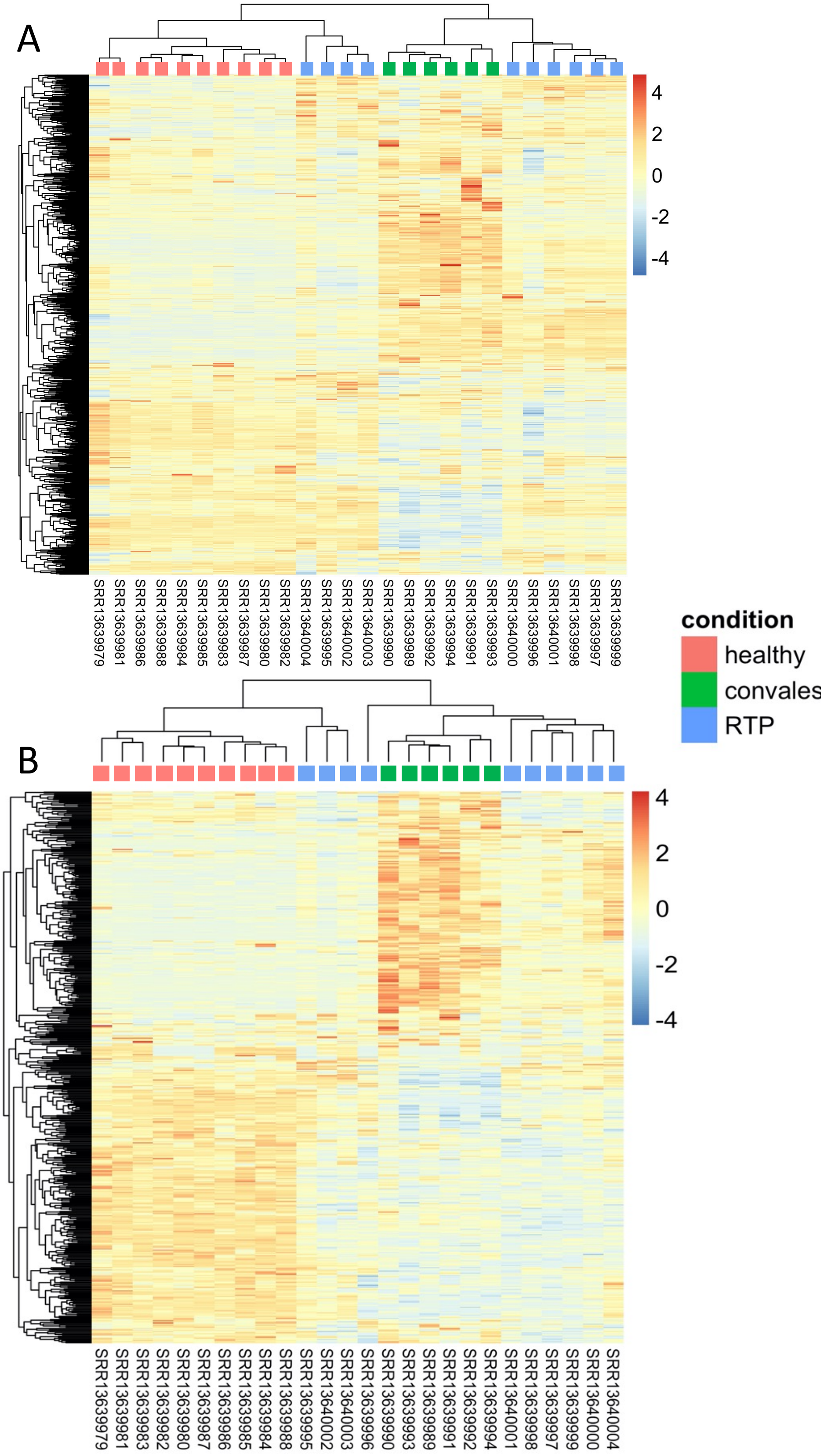

Supplementary Figure S6. Heatmap of the variance of expression for the subset of cellular genes involved in innate immunity

(A) Hierarchical clustering of a set of about 1100 cellular genes known to be involved in human innate immune response (as reported in InnateDB). The immune genes are in rows, and the samples are in columns. rlog-normalized counts are color-scaled from blue (minimum) to red (maximum). The heatmap has been compared to the one as obtained with the 500 HERVs sorted by the highest variance of expression among conditions (B) (already included in Figure 2, panel B). Correlation distance measure has been used in clustering columns. Samples are annotated by condition: red, healthy controls; green, convalescent after recover from SARS-CoV-2 infection; blue, re-testing positive after convalescence.

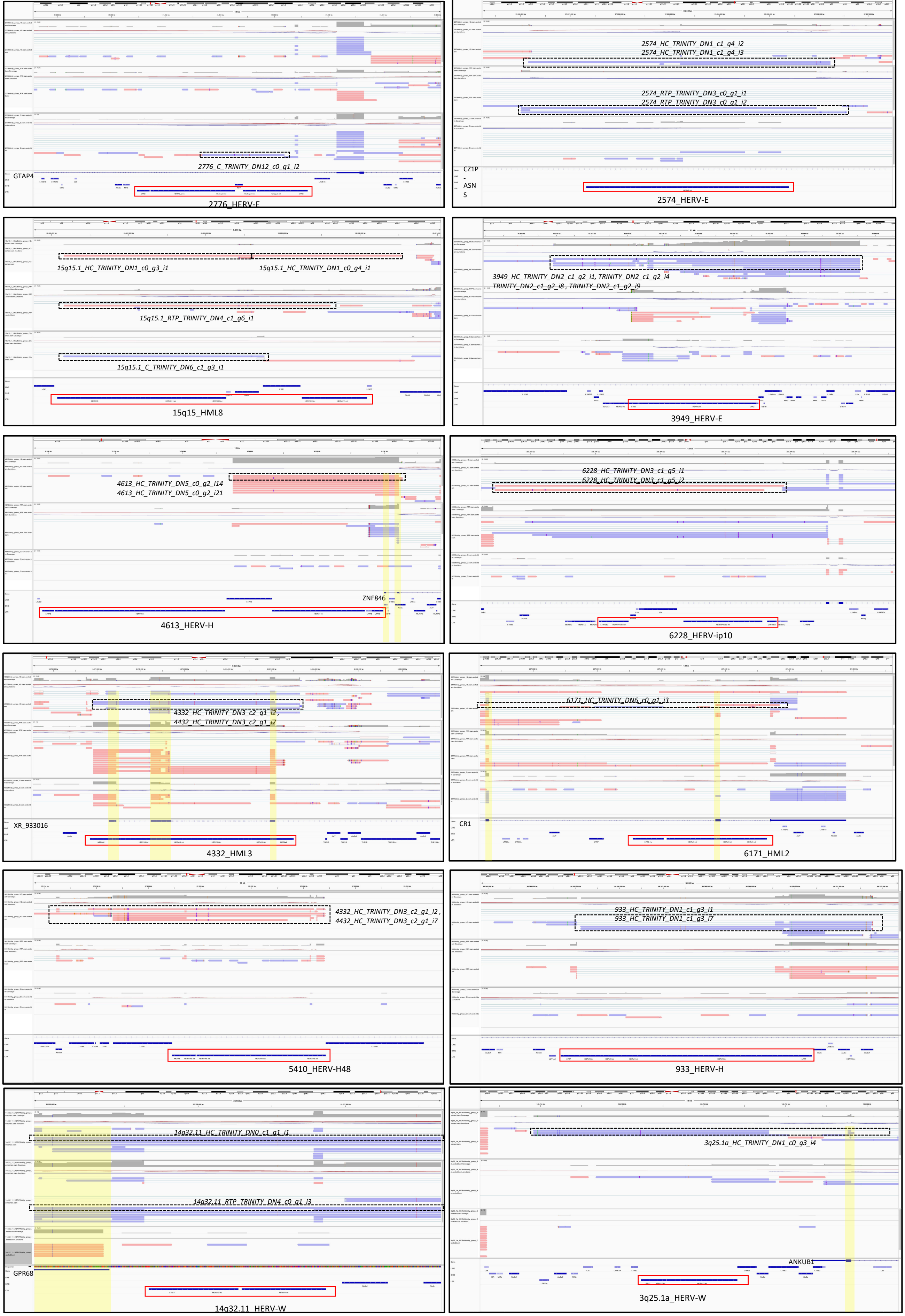

Supplementary Figure S7. Representation of the most relevant transcripts mapping to the key 31 deHERVs

HERV transcripts were reconstructed *de novo* using the Trinity pipeline from raw reads of the different HERV loci in the three conditions and are visualized at the correspondent position in the human genome with IGV software. Most relevant transcripts are included in a red square. Annotations for cellular genes ("Gene") and RepeatMasker classes of repetitive elements ("LINE", "SINE", and "LTR") were also activated from IGV tracks and are reported at the bottom of each panel. Overlap of the indicated transcripts with gene exons is highlighted in yellow boxes, indicating potential chimeric transcripts.

Supplementary Tables

Supplementary Table S1. Characteristics of the RNAseq dataset used for the study
The table reports each sample ID with the corresponding information, including raw fastq files reads' count and GC content, percentage and number of mapped reads by STAR alignment to hg38 assembly, percentage and number of reads counted at cellular genes and HERV genomic coordinates.

Supplementary Table S2. List of 282 deHERVs modulated in the PBMC of SARS-CoV-2 exposed individuals
The reported deHERVs loci were identified comparing together C and RTP individuals to HC with the package DEseq2m, setting as a threshold an absolute Log2FoldChange ≥ 1 and an adjusted p-value ≤ 0.01.

Supplementary Table S3. Cellular genes co-localized with deHERVs modulated in the PBMC of SARS-CoV-2 exposed individuals
The information of genes colocalized with deHERVs (including genomic coordinates, strand, exon count, and protein coding capacity) are reported along with the results of differential expression analysis in the same conditions (C and RTP vs HC).

Supplementary Table S4. Focus on the 50 de-genes co-localized with deHERVs modulated in the PBMC of SARS-CoV-2 exposed individuals
Results of differential expression analyses for deHERVs and co-localized de-genes are reported, indicating whether they show a concomitant modulation (in 49 out of 50 cases).

Supplementary Table S5. Results of HERV differential expression analyses in the different subcomparisons
After the overall analysis, the individual conditions have been pairwise compared in dedicated differential expression analyses of C vs HC (571 deHERVs), RTP vs HC (282 deHERVs), and RTP vs C (164 deHERVs). Modulation results are always referred to the first condition as compared to the second one.

Supplementary Table S6. Gene ontology of upregulated de-genes colocalized with upregulated de-HERV in the different subcomparisons
Based on the results reported in the previous table (Table S5), we identified genes co-localized with upregulated deHERVs and being themselves upregulated and performed a gene enrichment analysis using the database of GO biological processes on modEnrichr suite (https://maayanlab.cloud/modEnrichr/).

Supplementary Table S7. Expression levels of the 31 deHERVs modulated in all conditions
A total of 31 deHERVs found to be modulated in all subcomparisons (C vs HC, RTP vs HC, and RTP vs C) were further characterized by calculating their expression in the different samples as Transcripts per Million Kilobases (TPM).

Supplementary Table S8. De novo reconstruction of deHERVs putative transcripts
The 31 deHERVs found to be modulated in all conditions (Table S7) have been filtered based on a threshold of mean TPM > 2.5, selecting 12 deHERVs that have been characterized for their transcript production potential after the *de novo* reconstruction of C, RTP, and HC transcriptomes with the tool Trinity.
